# Supplementary material for: PARP14 inhibition restores PD-1 immune checkpoint inhibitor response following IFNγ-driven acquired resistance in preclinical cancer models
Source: Nat Commun. 2023 Sep 26;14:5983. doi: 10.1038/s41467-023-41737-1 (PMC10522711; doi:10.1038/s41467-023-41737-1)
Supplement: Supplementary file 1 — Supplementary Information [file 41467_2023_41737_MOESM1_ESM.pdf]

Supplementary Information

PARP14 inhibition restores PD-1 immune checkpoint inhibitor response following IFNγ-driven acquired resistance in preclinical cancer models

Chun Wai Wong[1,2], Christos Evangelou[1,2], Kieran N. Sefton[1,2,$], Rotem Leshem[1,2,$], Wei Zhang[1], Vishaka Gopalan[3], Sorayut Chattrakarn[1,2], Macarena Lucia Fernandez Carro[1,2], Erez Uzuner[1,2], Holly Mole[1], Daniel J. Wilcock[1], Michael P. Smith[1], Kleita Sergiou[1], Brian A. Telfer[1], Dervla T. Isaac[4], Chang Liu[4], Nicholas R. Perl[4], Kerrie Marie[1], Paul Lorigan[1,5], Kaye J. Williams[1], Patricia E. Rao[6], Raghavendar T. Nagaraju[1,7], Mario Niepel[4], and Adam F.L. Hurlstone[1,2,#]

[1]Faculty of Biology, Medicine and Health, The University of Manchester, Manchester, M13 9PT, UK

[2]Lydia Becker Institute of Immunology, The University of Manchester, Manchester, M13 9PT, UK

[3]Cancer Data Science Laboratory, National Cancer Institute, Bethesda, MD 20814, USA

[4]Ribon Therapeutics Inc., 35 Cambridge Park Drive, Suite 300, Cambridge, MA 02140, USA

[5]Department of Medical Oncology, The Christie NHS Foundation Trust, Wilmslow Road, Withington, Manchester M20 4BX, UK

[6]Patricia E. Rao Consulting, Acton, MA 01720, USA

[7]Colorectal and Peritoneal Oncology Centre, The Christie NHS Foundation Trust, Wilmslow Road, Withington, Manchester, UK

[$]These authors contributed equally

[#]To whom correspondence should be addressed: Dr Adam Hurlstone, Michael Smith Building, The University of Manchester, Dover Street, Manchester M13 9PT, UK; tel: +44 161 2751574; email: adam.hurlstone@manchester.ac.uk

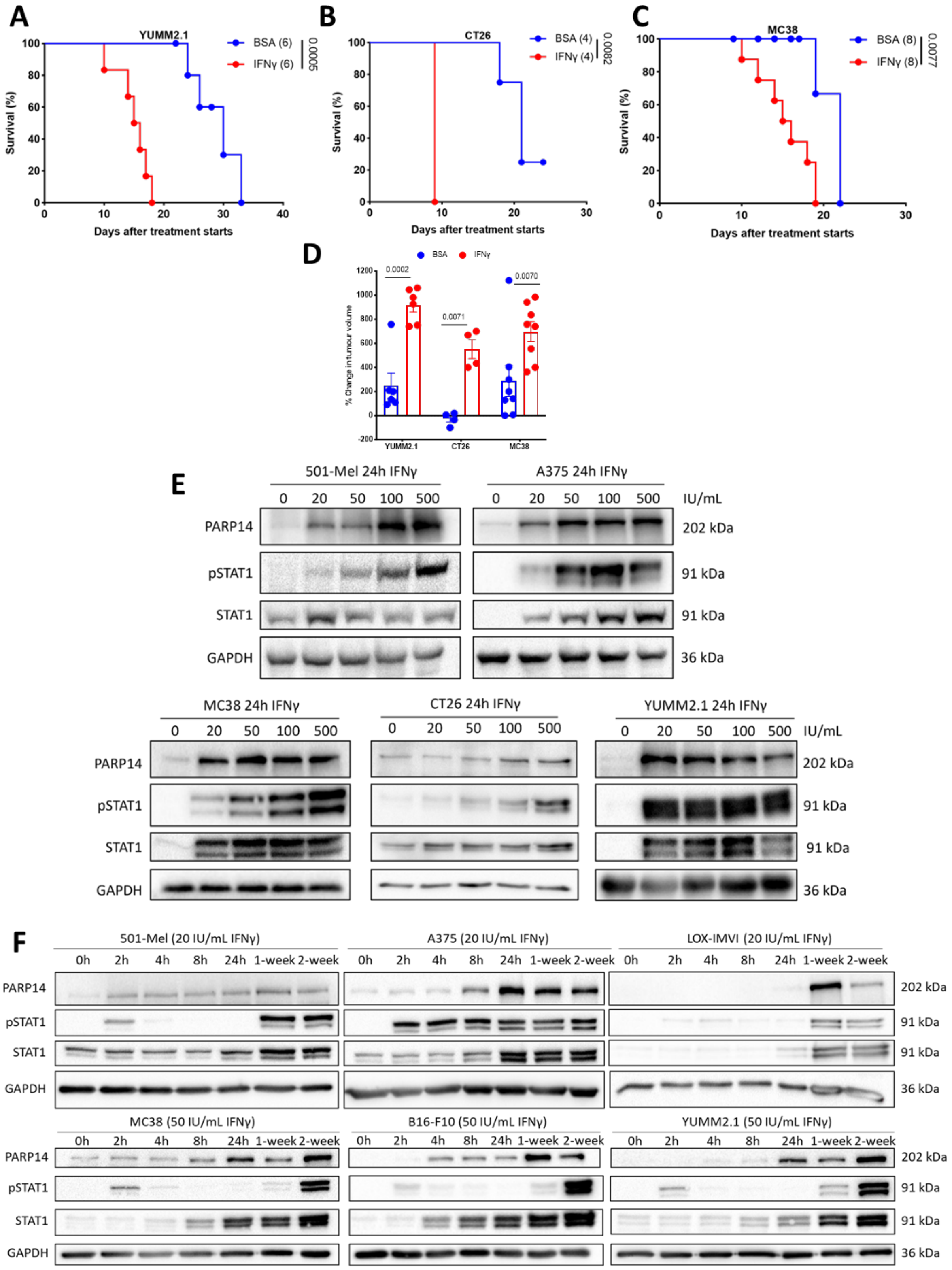
A
YUMM2.1
BSA (6)
IFNγ (6)
0.0005
Survival (%)
Days after treatment starts
B
CT26
BSA (4)
IFNγ (4)
0.0082
Survival (%)
Days after treatment starts
C
MC38
BSA (8)
IFNγ (8)
0.0077
Survival (%)
Days after treatment starts
D
BSA
IFNγ
0.0002
0.0071
0.0070
% Change in tumour volume
YUMM2.1
CT26
MC38
E
501-Mel 24h IFNγ
A375 24h IFNγ
0 20 50 100 500
IU/mL
PARP14
pSTAT1
STAT1
GAPDH
202 kDa
91 kDa
91 kDa
36 kDa
MC38 24h IFNγ
CT26 24h IFNγ
YUMM2.1 24h IFNγ
F
501-Mel (20 IU/mL IFNγ)
A375 (20 IU/mL IFNγ)
LOX-IMVI (20 IU/mL IFNγ)
0h 2h 4h 8h 24h 1-week 2-week
MC38 (50 IU/mL IFNγ)
B16-F10 (50 IU/mL IFNγ)
YUMM2.1 (50 IU/mL IFNγ)

Supplementary Figure 1. PARP14 and STAT1 activation are similarly upregulated in response to IFNγ. Continuing with experimental setup in Figure 1A, Kaplan-Meier survival plots of each animal receiving (A) YUMM2.1 (BSA: n = 6; IFNγ: n = 6), (B) CT26 (BSA: n = 4; IFNγ: n = 4), and (C) MC38 (BSA: n = 8; IFNγ: n = 8) implants with 2-week pretreatment of IFNγ or BSA. The p-values were determined by Log-rank (Mantel- Cox) test. (D) The percentage tumour volume change between the start of treatment and one week after the last α-PD-1 dose in YUMM2.1 (BSA: n = 6; IFNγ: n = 6) and MC38 (BSA: n = 8; IFNγ: n = 8) and the day that final dose was administered in CT26 (BSA: n = 4; IFNγ: n = 4). The data were presented as mean ± S.E.M. and the adjusted p-values were assessed by two-way ANOVA Šídák's test. (E) 501-MEL (n = 3), A375 (n = 3), MC38 (n = 3), CT26 (n = 3), and YUMM.2.1 (n = 3) tumour cell lines treated for 24 hours with increasing concentrations of IFNγ (0, 20, 50, 100 and 500 IU/mL), with PARP14, pSTAT1 and STAT1 protein expression determined by western blot relative to a GAPDH loading control. The images were representatives of 1 of 3 independent experiments. (F) Treatment of human (501-MEL (n = 3), A375 (n = 3), and LOX-IMVI (n = 3)) or mouse (MC38 (n = 3), B16-F10 (n = 3), and YUMM2.1 (n = 3)) tumour cell lines with 20 IU/mL (human) or 50 IU/mL (mouse) IFNγ for up to 2 weeks. PARP14, pSTAT1 and STAT1 protein expression were determined via western blot relative to a GAPDH loading control. The images were representatives of 1 of 3 independent experiments. Source data are provided as a Source Data file.

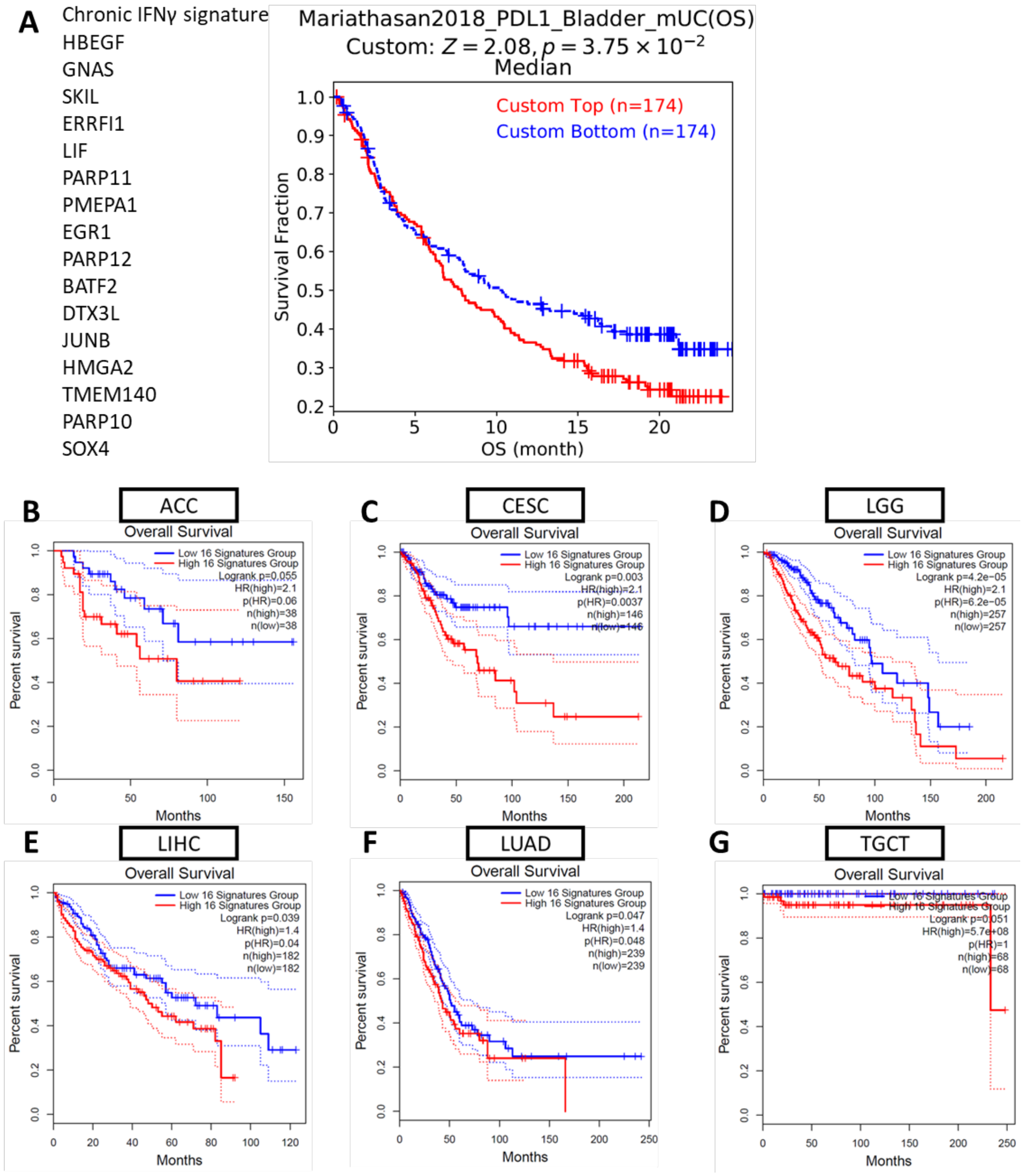

A
Chronic IFNγ signature
HBEGF
GNAS
SKIL
ERRFI1
LIF
PARP11
PMEPA1
EGR1
PARP12
BATF2
DTX3L
JUNB
HMGA2
TMEM140
PARP10
SOX4
Mariathasan2018_PDL1_Bladder_mUC(OS)
Custom: Z = 2.08, p = 3.75 × 10^−2
Median
Custom Top (n=174)
Custom Bottom (n=174)
Survival Fraction
OS (month)
B
ACC
Overall Survival
Low 16 Signatures Group
High 16 Signatures Group
Logrank p=0.055
HR(high)=2.1
p(HR)=0.06
n(high)=38
n(low)=38
Percent survival
Months
C
CESC
Overall Survival
Low 16 Signatures Group
High 16 Signatures Group
Logrank p=0.003
HR(high)=2.1
p(HR)=0.0037
n(high)=146
n(low)=146
Percent survival
Months
D
LGG
Overall Survival
Low 16 Signatures Group
High 16 Signatures Group
Logrank p=4.2e−05
HR(high)=2.1
p(HR)=6.2e−05
n(high)=257
n(low)=257
Percent survival
Months
E
LIHC
Overall Survival
Low 16 Signatures Group
High 16 Signatures Group
Logrank p=0.039
HR(high)=1.4
p(HR)=0.04
n(high)=182
n(low)=182
Percent survival
Months
F
LUAD
Overall Survival
Low 16 Signatures Group
High 16 Signatures Group
Logrank p=0.047
HR(high)=1.4
p(HR)=0.048
n(high)=239
n(low)=239
Percent survival
Months
G
TGCT
Overall Survival
Low 16 Signatures Group
High 16 Signatures Group
Logrank p=0.051
HR(high)=5.7e+08
p(HR)=1
n(high)=68
n(low)=68
Percent survival
Months

Supplementary Figure 2. Chronic IFNγ gene signature derived from YUMM2.1 and CT26 tumour cells predicts worse survival in patients. (A) A sixteen-gene chronic IFNγ signature was developed by identifying genes commonly and selectively upregulated in YUMM2.1 and CT26 cells exposed continuously for 3 weeks to IFNγ (see methods section for details). Kaplan-Meier survival analysis, comparing patients with metastatic urothelial cancer who received anti-PD-L1 agent (atezolizumab)[1] stratified by median chronic IFNγ signature score, revealed significantly worse survival in the higher scoring group. Similarly, stratifying as above and performing Kaplan-Meier survival analysis using data for multiple TCGA cancer types revealed worse overall survival in higher scoring patients with (B) Adrenocortical carcinoma (ACC), (C) Cervical squamous cell carcinoma and endocervical adenocarcinoma (CESC), (D) Brain Lower Grade Glioma (LGG), (E) Liver hepatocellular carcinoma (LIHC), (F) Lung adenocarcinoma (LUAD), and (G) Testicular Germ Cell Tumors (TGCT).

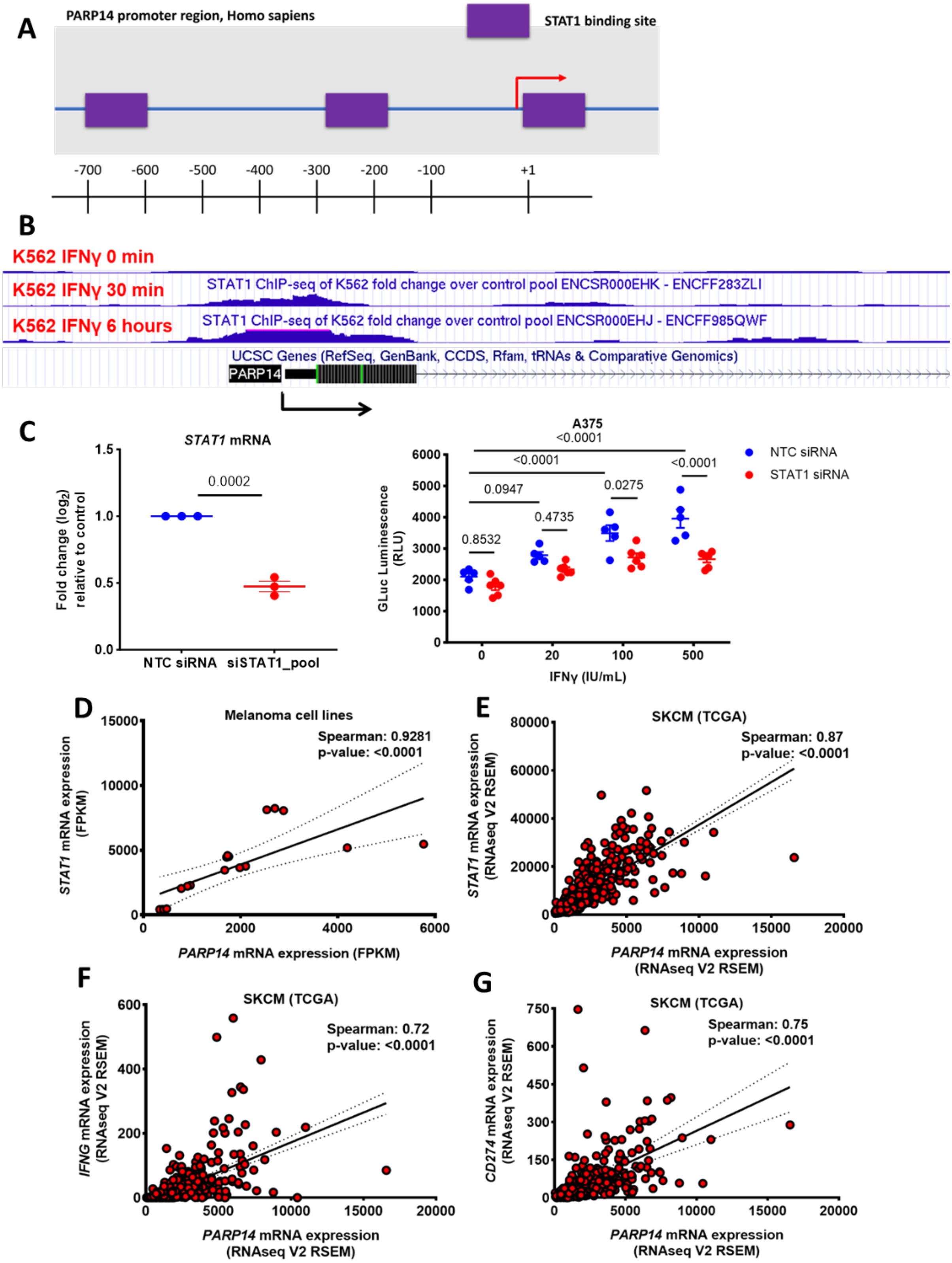
A
PARP14 promoter region, Homo sapiens
STAT1 binding site
-700
-600
-500
-400
-300
-200
-100
+1
B
K562 IFNγ 0 min
K562 IFNγ 30 min
K562 IFNγ 6 hours
STAT1 ChIP-seq of K562 fold change over control pool ENCSR000EHK - ENCFF283ZLI
STAT1 ChIP-seq of K562 fold change over control pool ENCSR000EHJ - ENCFF985QWF
UCSC Genes (RefSeq, GenBank, CCDS, Rfam, tRNAs & Comparative Genomics)
PARP14
C
STAT1 mRNA
Fold change (log2) relative to control
0.0002
NTC siRNA
siSTAT1_pool
A375
GLuc Luminescence (RLU)
IFNγ (IU/mL)
NTC siRNA
STAT1 siRNA
<0.0001
0.0947
0.4735
0.0275
0.8532
D
Melanoma cell lines
Spearman: 0.9281
p-value: <0.0001
STAT1 mRNA expression (FPKM)
PARP14 mRNA expression (FPKM)
E
SKCM (TCGA)
Spearman: 0.87
p-value: <0.0001
STAT1 mRNA expression (RNAseq V2 RSEM)
PARP14 mRNA expression (RNAseq V2 RSEM)
F
SKCM (TCGA)
Spearman: 0.72
p-value: <0.0001
IFNG mRNA expression (RNAseq V2 RSEM)
PARP14 mRNA expression (RNAseq V2 RSEM)
G
SKCM (TCGA)
Spearman: 0.75
p-value: <0.0001
CD274 mRNA expression (RNAseq V2 RSEM)
PARP14 mRNA expression (RNAseq V2 RSEM)

Supplementary Figure 3. STAT1 is required for IFNγ-mediated PARP14 induction, and PARP14 and STAT1 expression strongly correlate in melanoma cell lines and patients. (A) Schematic indicating positions of putative STAT1 binding sites (purple boxes) in the upstream PARP14 promoter region (~0.8 kb), as identified with MatInspector in silico analysis. The transcriptional start site is indicated by a red arrow. (B) ChIP-seq data analysis (data retrieved from ENCODE GSM935487 Snyder lab 2010) also verified the binding of STAT1 near the transcription start site of PARP14. (C) A375 cells were transfected with a GLuc reporter plasmid in which luciferase expression is controlled by the PARP14 promoter, and then treated with either a pool of siRNA targeting STAT1 (n = 6) or a NTC (n = 5) siRNA construct. Lefthand plot shows RT-qPCR analysis of *STAT1* mRNA expression for A375 cells (NTC: n = 3; STAT1: n = 3) upon treatment with each siRNA. The data was presented as mean ± S.E.M. and the p-value was determined by two-tailed unpaired t test. Righthand plot showed luciferase activity in response to 24-hour treatment with a range of IFNγ doses (0, 20, 100 and 500 IU/mL) in cells treated with either of the two siRNAs. Luminescence values were normalised to the cell confluency of each respective group, as determined by staining with crystal violet. The lefthand data was presented as mean ± S.D. and the p-value was determined by two-tailed unpaired t test; the righthand data were presented as mean ± S.E.M. and the adjusted p-values were determined by two-way ANOVA Tukey's test. (D–G) mRNA expression scatter plots showing co-expression of (D) *PARP14* with *STAT1* in melanoma cell lines (n = 17) or of *PARP14* with (E) *STAT1* (n = 472), (F) *IFNG* (n = 472), and (G) *CD274* (n = 472) in melanoma (SKCM, TCGA) patients. Spearman correlations and P-values were shown. Source data are provided as a Source Data file.

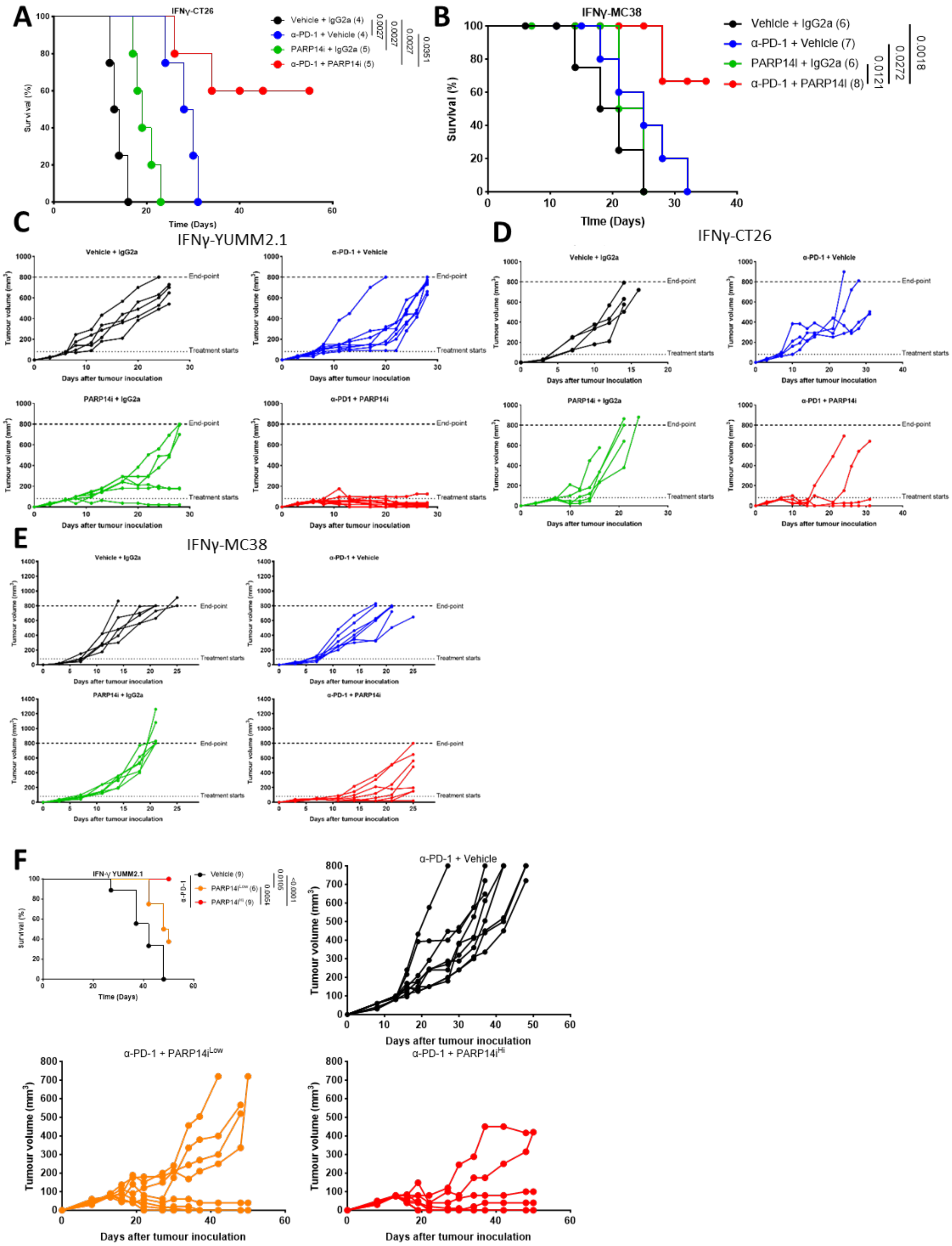
A
IFNγ-CT26
Vehicle + IgG2a (4)
α-PD-1 + Vehicle (4)
PARP14i + IgG2a (5)
α-PD-1 + PARP14i (5)
0.0027
0.0027
0.0027
0.0351
Survival (%)
Time (Days)
B
IFNγ-MC38
Vehicle + IgG2a (6)
α-PD-1 + Vehicle (7)
PARP14i + IgG2a (6)
α-PD-1 + PARP14i (8)
0.0121
0.0272
0.0018
Survival (%)
Time (Days)
C
IFNγ-YUMM2.1
Vehicle + IgG2a
α-PD-1 + Vehicle
PARP14i + IgG2a
α-PD1 + PARP14i
End-point
Treatment starts
Tumour volume (mm³)
Days after tumour inoculation
D
IFNγ-CT26
Vehicle + IgG2a
α-PD-1 + Vehicle
PARP14i + IgG2a
α-PD1 + PARP14i
End-point
Treatment starts
Tumour volume (mm³)
Days after tumour inoculation
E
IFNγ-MC38
Vehicle + IgG2a
α-PD-1 + Vehicle
PARP14i + IgG2a
α-PD-1 + PARP14i
End-point
Treatment starts
Tumour volume (mm³)
Days after tumour inoculation
F
IFN-γ YUMM2.1
Vehicle (9)
PARP14i^Low (6)
PARP14i^Hi (9)
α-PD-1
0.0054
0.0105
<0.0001
Survival (%)
Time (Days)
α-PD-1 + Vehicle
α-PD-1 + PARP14i^Low
α-PD-1 + PARP14i^Hi
Tumour volume (mm³)
Days after tumour inoculation

Supplementary Figure 4. Pharmacological inhibition of PARP14 delays relapse of chronic IFNγ-treated tumours when combined with α-PD1 therapy. (A–B) Kalan-Meier plots of mice receiving IFNγ pre-treated (A) CT26 (Vehicle + IgG2a: n = 4; α-PD-1 + Vehicle: n = 4; PARP14i + IgG2a: n = 5; α-PD-1 + PARP14i: n = 5) and (B) MC38 (Vehicle + IgG2a: n = 6; α-PD-1 + Vehicle: n = 7; PARP14i + IgG2a: n = 6; α-PD-1 + PARP14i: n = 8) implants in the experiment shown in Figure 3A. The p-values were determined by Log-rank (Mantel-Cox) test. (C-E) Growth rate for each tumour derived from IFNγ pre-treated (C) YUMM2.1 (Vehicle + IgG2a: n = 5; α-PD-1 + Vehicle: n = 8; PARP14i + IgG2a: n = 6; α-PD-1 + PARP14i: n = 12), (D) CT26 (Vehicle + IgG2a: n = 4; α-PD-1 + Vehicle: n = 4; PARP14i + IgG2a: n = 5; α-PD-1 + PARP14i: n = 5), (E) MC38 (Vehicle + IgG2a: n = 6; α-PD-1 + Vehicle: n = 7; PARP14i + IgG2a: n = 6; α-PD-1 + PARP14i: n = 8). (F) Chronic IFNγ pre-treated YUMM2.1 cells were subcutaneously implanted into 8–12-week-old wild-type syngeneic female mice. Treatment with either α-PD-1 or IgG2a antibody was initiated once tumour volume reached 80-100 mm^3, with antibodies administered every three days for a total of four doses. In parallel, the animals received two daily doses of vehicle or three different concentrations of PARP14i RBN012759: 0 mg/Kg denoted as Vehicle (n = 9), 50 mg/Kg denoted as $PARP14i^{Low}$ (n = 6), and 500 mg/Kg denoted as $PARP14^{Hi}$ (n = 9) for a total of three weeks. For Kaplan-Meier plots the number of mice used per treatment arm was indicated in parenthesis and the p-values were determined by Log-rank (Mantel-Cox) test. Growth rate for each tumour were also shown per treatment arm. Source data are provided as a Source Data file.

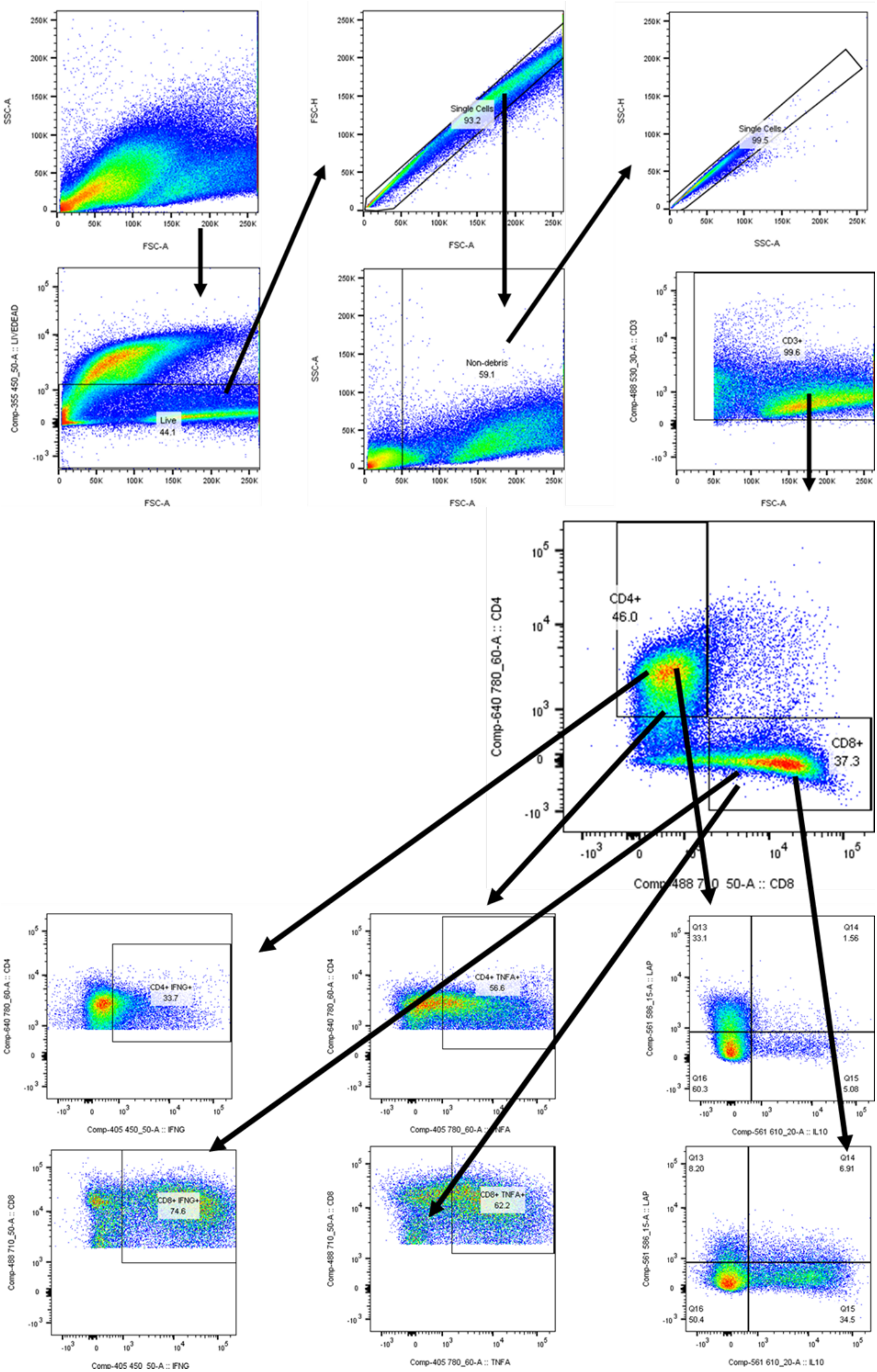

SSC-A
FSC-A
Comp-355 450_50-A :: LIVEDEAD
Live
44.1
FSC-H
Single Cells
93.2
Non-debris
59.1
SSC-H
Single Cells
99.5
Comp-488 530_30-A :: CD3
CD3+
99.6
Comp-640 780_60-A :: CD4
CD4+
46.0
CD8+
37.3
Comp-488 710_50-A :: CD8
CD4+ IFNG+
33.7
Comp-405 450_50-A :: IFNG
CD4+ TNFA+
56.6
Comp-405 780_60-A :: TNFA
Q13
33.1
Q14
1.56
Q16
60.3
Q15
5.08
Comp-561 586_15-A :: LAP
Comp-561 610_20-A :: IL10
CD8+ IFNG+
74.6
CD8+ TNFA+
62.2
Q13
8.20
Q14
6.91
Q16
50.4
Q15
34.5

Supplementary Figure 5. Flow cytometry gating strategy for assessing the influence of PARP14 pharmacological inhibition on cytokine production. Dead cells, debris and duplets were excluded, followed by gating for CD3. Live CD3+ cells were then gated to quantify CD4+ T cells and CD8+ T cells, which were then analysed for intracellular expression of IFNG, TNFA, LAP, and IL-10. Gates were set using FMO controls.

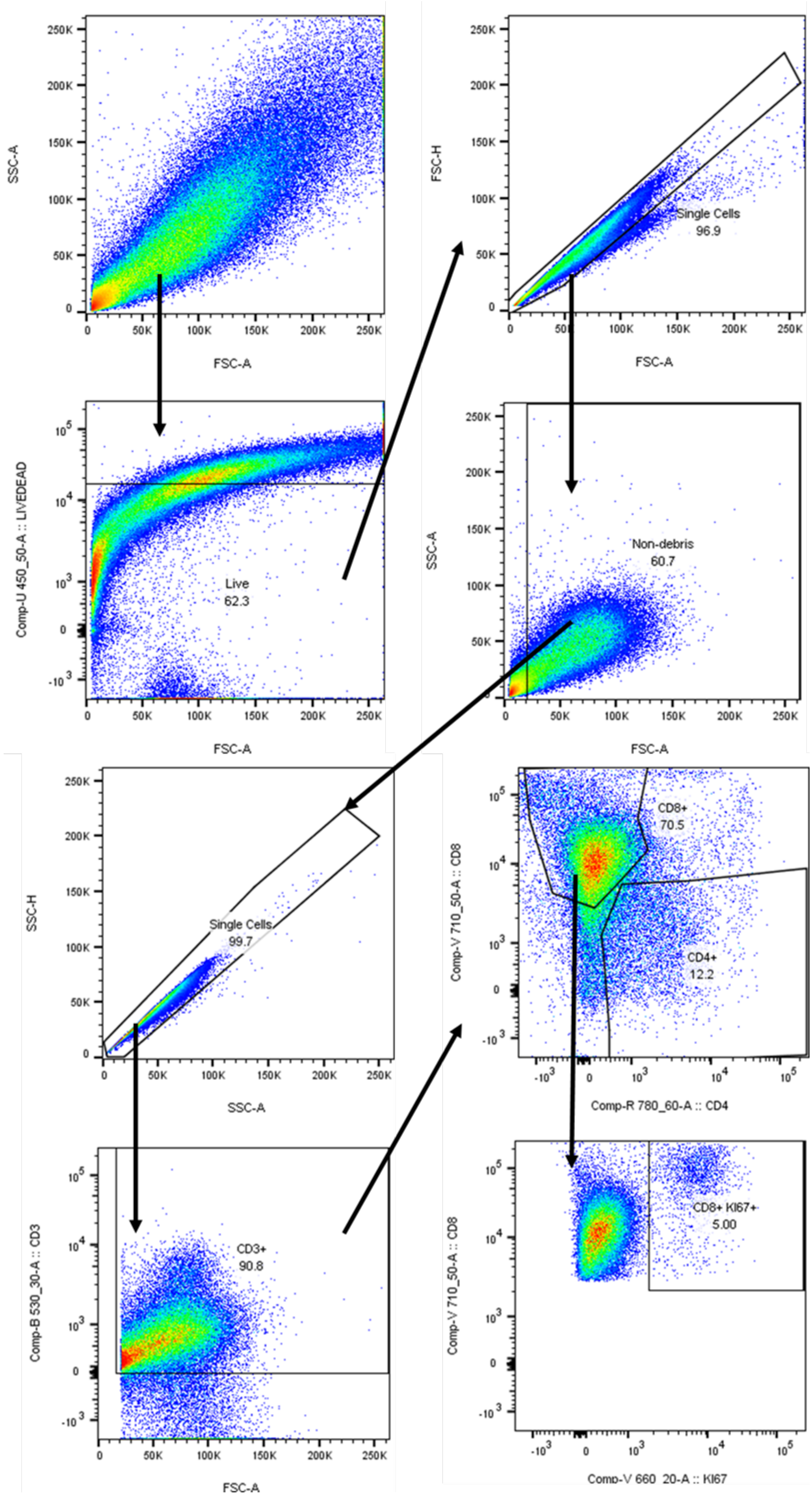

SSC-A
FSC-A
Comp-U 450_50-A :: LIVEDEAD
Live
62.3
FSC-H
Single Cells
96.9
Non-debris
60.7
SSC-H
SSC-A
Single Cells
99.7
Comp-B 530_30-A :: CD3
CD3+
90.8
Comp-V 710_50-A :: CD8
Comp-R 780_60-A :: CD4
CD8+
70.5
CD4+
12.2
Comp-V 660_20-A :: KI67
CD8+ KI67+
5.00

Supplementary Figure 6. Flow cytometry gating strategy for assessing the influence of PARP14 pharmacological inhibition on intracellular expression of Ki67 in CD8+ T cells. Dead cells, debris and duplets were excluded, followed by gating for CD3. Live CD3+ cells were then gated to quantify CD4+ T cells and CD8+ T cells, which were then analysed for intracellular expression of Ki-67. Gates were set using FMO controls.

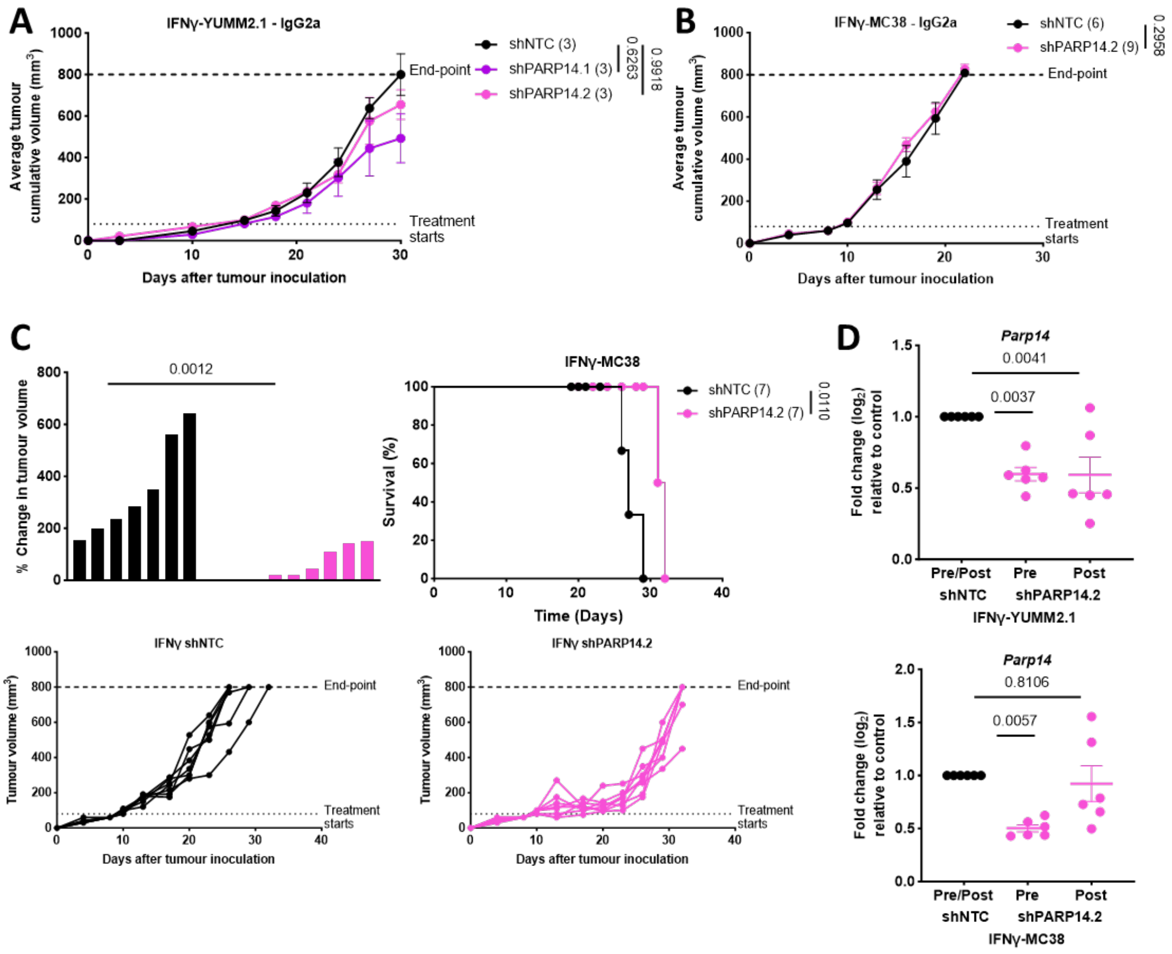

A
IFNγ-YUMM2.1 - IgG2a
Average tumour cumulative volume (mm3)
Days after tumour inoculation
shNTC (3)
shPARP14.1 (3)
shPARP14.2 (3)
0.6263
0.9918
End-point
Treatment starts
B
IFNγ-MC38 - IgG2a
shNTC (6)
shPARP14.2 (9)
0.2958
End-point
Treatment starts
Average tumour cumulative volume (mm3)
Days after tumour inoculation
C
% Change in tumour volume
0.0012
IFNγ-MC38
Survival (%)
Time (Days)
shNTC (7)
shPARP14.2 (7)
0.0110
IFNγ shNTC
IFNγ shPARP14.2
Tumour volume (mm3)
Days after tumour inoculation
End-point
Treatment starts
D
Parp14
0.0041
0.0037
Fold change (log2) relative to control
Pre/Post
Pre
Post
shNTC
shPARP14.2
IFNγ-YUMM2.1
Parp14
0.8106
0.0057
Fold change (log2) relative to control
Pre/Post
Pre
Post
shNTC
shPARP14.2
IFNγ-MC38

Supplementary Figure 7. PARP14 depletion promotes α-PD-1 response in tumours derived from IFNγ-pre-treated MC38 cells. (A–B) Average cumulative tumour volume growth curves for (A) YUMM2.1 (shNTC: n = 3; shPARP14.1: n = 3; shPARP14.2: n = 3) and (B) MC38 (shNTC: n = 6; shPARP14.2: n = 9) tumours, expressing control (shNTC) and PARP14-targeting (shPARP14) shRNA treated with control IgG2a. The data were presented as mean ± S.E.M. and the adjusted p-values were assessed for the last day of IgG2a treatment by one-way ANOVA Dunnett's test in YUMM2.1 and two-tailed unpaired t test in MC38. (C) Percentage tumour volume change between the first dose of antibody treatment and administration of the final α-PD-1 dose (top left), corresponding Kalan-Meier plots (top right), and individual tumour growth rates (bottom) for the experimental protocol detailed in Figure 4A (IFNγ-MC38 shNTC: n = 7 and IFNγ-MC38 shPARP14.2: n = 7). The p-values were assessed by two-tailed unpaired t test for the percentage tumour volume change and Log-rank (Mantel-Cox) test for the survival plot. (D) RT-qPCR analysis of *Parp14* mRNA expression pre- or post-tumour implantation for IFNγ-YUMM2.1 (shNTC: n = 6; shPARP14.2: n = 6) and IFNγ-MC38 (shNTC: n = 6; shPARP14.2: n = 6). The data were presented as mean ± S.E.M. and the adjusted p-values were determined by one-way ANOVA Dunnett's test. Source data are provided as a Source Data file.

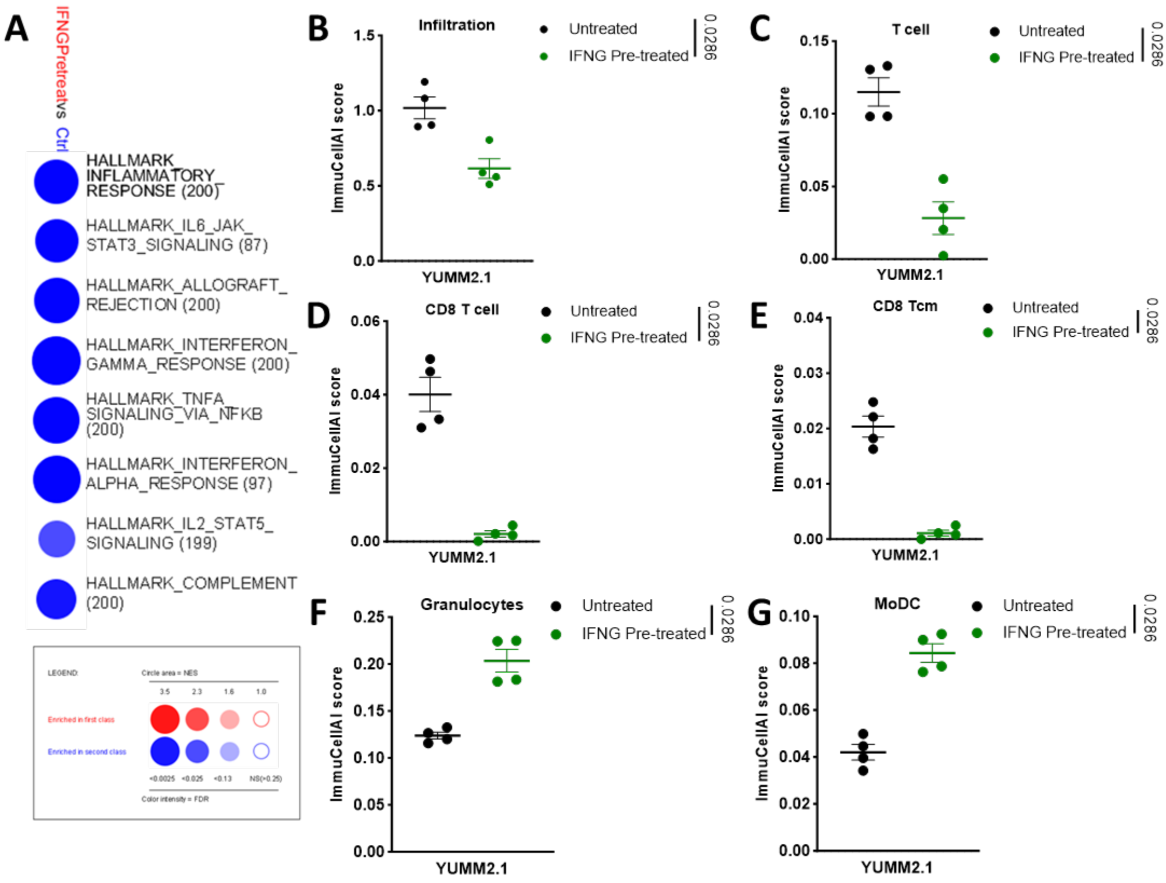

A
IFNGPretreat vs Ctrl
HALLMARK_INFLAMMATORY_RESPONSE (200)
HALLMARK_IL6_JAK_STAT3_SIGNALING (87)
HALLMARK_ALLOGRAFT_REJECTION (200)
HALLMARK_INTERFERON_GAMMA_RESPONSE (200)
HALLMARK_TNFA_SIGNALING_VIA_NFKB (200)
HALLMARK_INTERFERON_ALPHA_RESPONSE (97)
HALLMARK_IL2_STAT5_SIGNALING (199)
HALLMARK_COMPLEMENT (200)
B
Infiltration
Untreated
IFNG Pre-treated
0.0286
ImmuCellAI score
YUMM2.1
C
T cell
D
CD8 T cell
E
CD8 Tcm
F
Granulocytes
G
MoDC

Supplementary Figure 8. Tumours derived from chronic IFNγ pre-treated tumour cells are immunologically 'cold'. (A) GSEA based on RNA-seq data depicting hallmark processes enriched in two-week chronic IFNγ pre-treated tumours (n = 4) versus BSA-treated (n = 4) tumours, which were treated with IgG2a antibodies. Circle area depicts the NES, and colour intensity depicts the FDR, with ≤ 0.25 classed as significant. (B–G) Bulk-tumour RNA-seq results derived from Untreated (n = 4) and IFNG Pre-treated (n = 4) analysed by cell type enrichment analysis (ImmuCellAI), with scores shown for (B) infiltration, (C) T cell, (D) CD8 T cell, (E) CD8 Tcm, (F) Granulocytes, and (G) Myeloid Dendritic cell (MoDC). The data were presented as mean ± S.E.M. and the p-values were determined by unpaired two-tailed Mann- Whitney test. Source data are provided as a Source Data file.

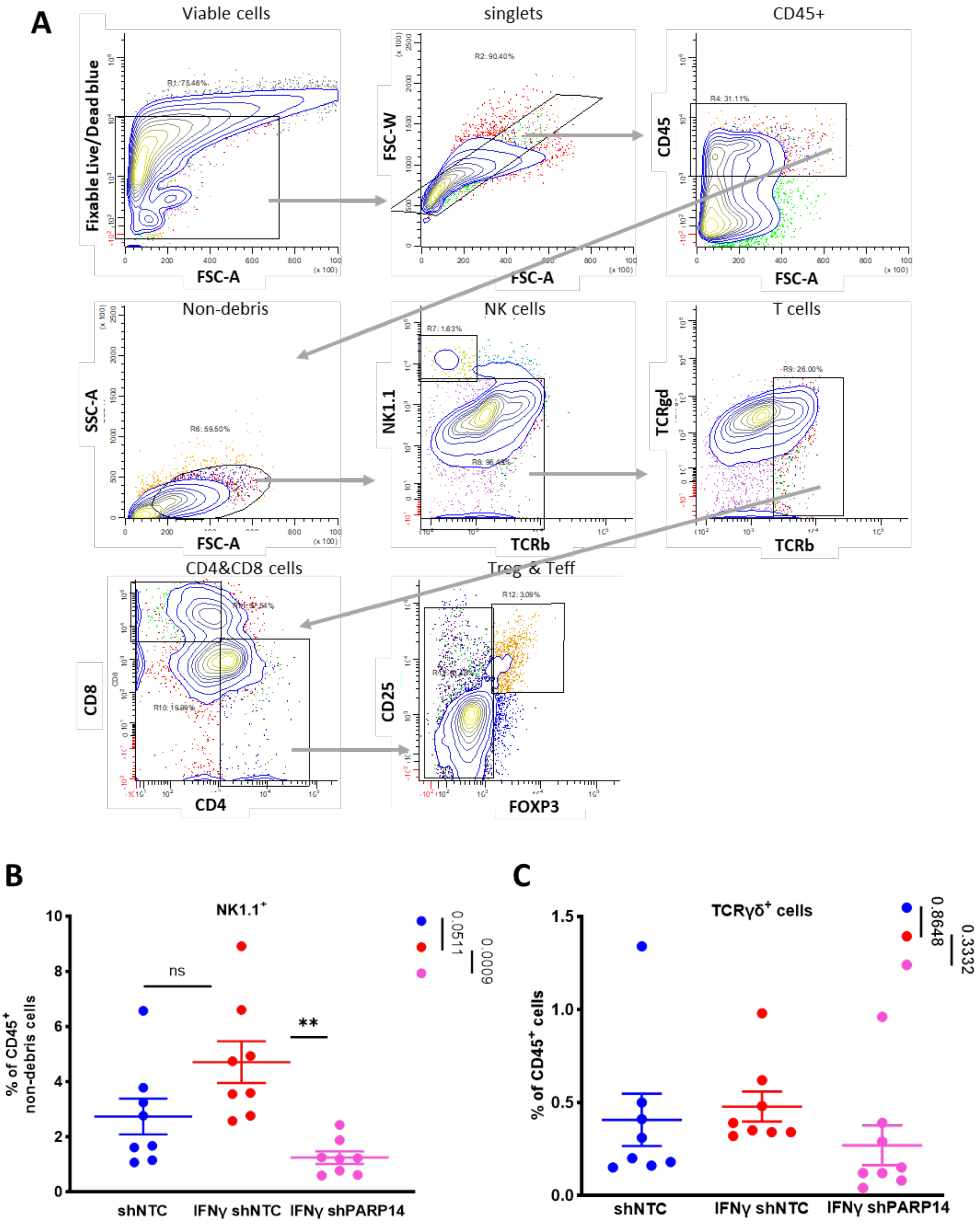
A
Viable cells
singlets
CD45+
Fixable Live/Dead blue
FSC-A
FSC-W
FSC-A
CD45
FSC-A
Non-debris
NK cells
T cells
SSC-A
FSC-A
NK1.1
TCRb
TCRgd
TCRb
CD4&CD8 cells
Treg & Teff
CD8
CD4
CD25
FOXP3
B
NK1.1+
% of CD45+ non-debris cells
ns
**
0.0511
0.0009
shNTC
IFNγ shNTC
IFNγ shPARP14
C
TCRγδ+ cells
% of CD45+ cells
0.8648
0.3332
shNTC
IFNγ shNTC
IFNγ shPARP14

Supplementary Figure 9. Flow cytometry gating strategy to assess the effects of sustained IFNγ exposure and PARP14 genetic depletion on tumour immune infiltrates. (A) Viable, singlet leukocytes (CD45+), after removal of small debris, gated using the markers NK1.1 TCRβ, TCRγδ, CD4, CD8 and FOXP3 to quantify NK cells, γδ T cells, TCRαβ T cells, CD4+ effector T cells, Tregs and CD8+ T cells. Gates were set using FMO controls. (B–C) 8–12-week-old wild-type C57BL/6 mice were subcutaneously implanted with IFNγ-naïve YUMM2.1 cells expressing shNTC (n = 8) or chronic IFNγ pre-treated YUMM2.1 cells expressing shNTC (n = 8) or shPARP14 (n = 8). Tumours were allowed to grow to 300-400 mm^3 and then dissected and disaggregated for analysis by flow cytometry. Populations of NK1.1+ cells (B) and TCRγδ+ cells (C) in the tumour infiltrate. The data were presented as mean ± S.E.M and the adjusted p-values were assessed by one-way ANOVA Dunnett's test. Source data are provided as a Source Data file.

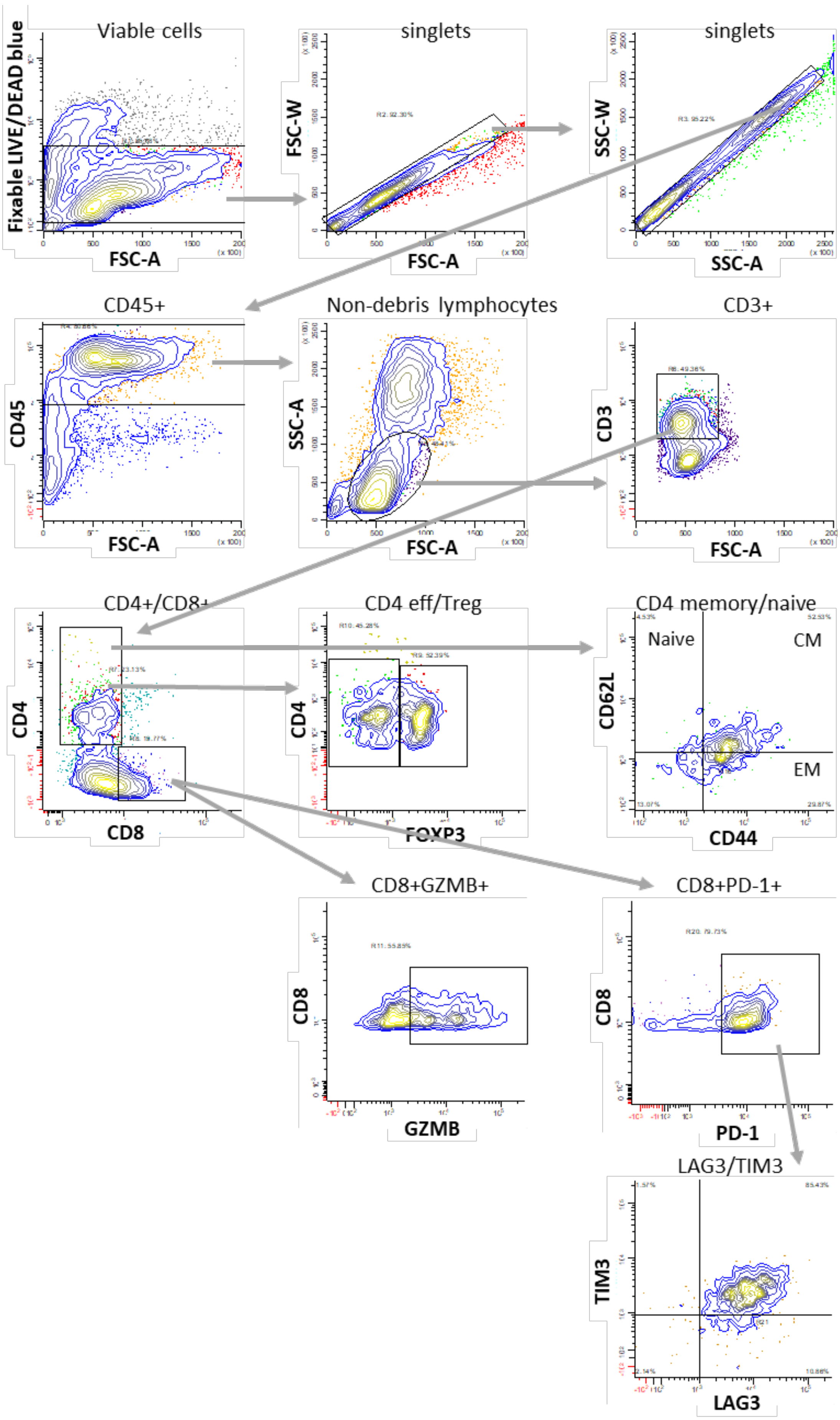

Viable cells
Fixable LIVE/DEAD blue
FSC-A
singlets
FSC-W
FSC-A
singlets
SSC-W
SSC-A
CD45+
CD45
FSC-A
Non-debris lymphocytes
SSC-A
FSC-A
CD3+
CD3
FSC-A
CD4+/CD8+
CD4
CD8
CD4 eff/Treg
CD4
FOXP3
CD4 memory/naive
Naive
CM
EM
CD62L
CD44
CD8+GZMB+
CD8
GZMB
CD8+PD-1+
CD8
PD-1
LAG3/TIM3
TIM3
LAG3

Supplementary Figure 10. Flow cytometry gating strategy for assessing the influence of PARP14 pharmacological inhibition on tumour infiltrating immune cells. Dead cells, debris and duplets were excluded and immune cells gated by CD45. Live CD45+ cells were then gated to quantify CD4+ helper T cells, Treg cells and CD8 T cells, which were then analysed for surface expression and intracellular expression of PD-1, LAG-3, TIM-3, and GZMB. Gates were set using FMO controls.

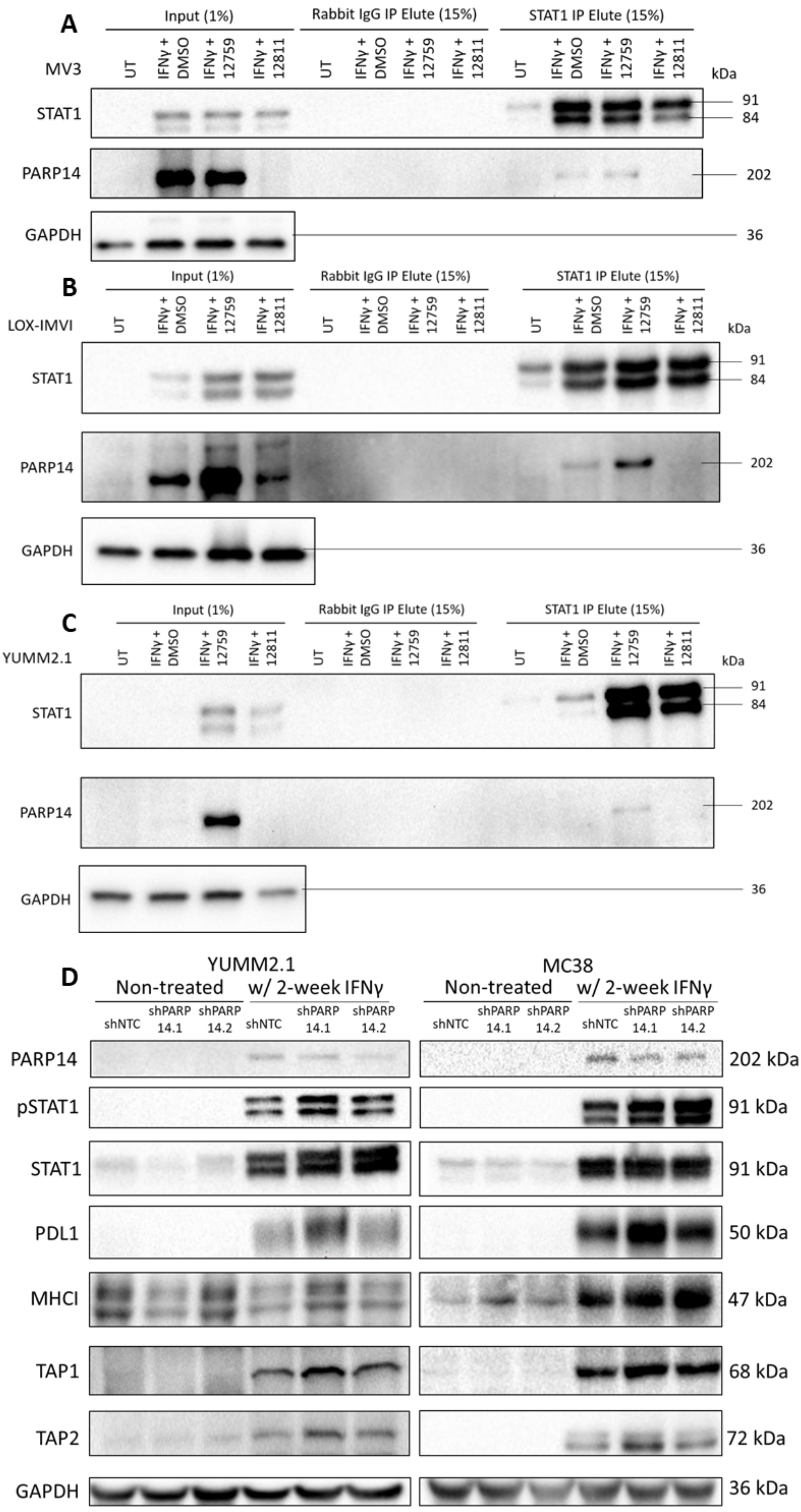
A
Input (1%)
Rabbit IgG IP Elute (15%)
STAT1 IP Elute (15%)
MV3
UT
IFNγ + DMSO
IFNγ + 12759
IFNγ + 12811
kDa
STAT1
PARP14
GAPDH
91
84
202
36
B
LOX-IMVI
C
YUMM2.1
D
YUMM2.1
MC38
Non-treated
w/ 2-week IFNγ
shNTC
shPARP 14.1
shPARP 14.2
pSTAT1
PDL1
MHCI
TAP1
TAP2
202 kDa
91 kDa
50 kDa
47 kDa
68 kDa
72 kDa
36 kDa

Supplementary Figure 11. STAT1 interacts with PARP14 in melanoma cells. Proteins were pulled down by immunoprecipitation with control or anti-STAT1 antibody from extracts of untreated, two-week chronic IFN-γ- plus 48-hour DMSO-treated, two-week chronic IFN-γ- plus 48-hour PARP14 catalytic inhibitor (12759)-treated, or two-week chronic IFN-γ- plus 48-hour PARP14 PROTAC (12811)-treated (A) MV3 (Untreated: n = 3; IFNγ + DMSO: n = 3; IFNγ + 12759: n = 3; IFNγ + 12811: n = 3; The images were representatives of 1 of 3 independent experiments), (B) LOX-IMVI (Untreated: n = 3; IFNγ + DMSO: n = 3; IFNγ + 12759: n = 3; IFNγ + 12811: n = 3; The images were representatives of 1 of 3 independent experiments), and (C) YUMM2.1 (Untreated: n = 3; IFNγ + DMSO: n = 3; IFNγ + 12759: n = 3; IFNγ + 12811: n = 3; The images were representatives of 1 of 3 independent experiments) tumour cells. STAT1 and PARP14 proteins were probed by western blot in input material (1% of total lysate), rabbit IgG IP samples (15% of total lysate), and STAT1 IP samples (15% of total lysate). GAPDH was used as a loading control in input samples. (D) Western blot analysis of levels of PARP14, STAT1, and STAT1 target gene products in YUMM2.1 (Non-treated shNTC: n = 3; Non-treated shPARP14.1: n = 3; Non-treated shPARP14.2: n = 3; w/ 2-week IFNγ shNTC: n = 3; w/ 2-week IFNγ shPARP14.1: n = 3; w/ 2-week IFNγ shPARP14.2: n = 3) and MC38 cells (Non-treated shNTC: n = 3; Non-treated shPARP14.1: n = 3; Non-treated shPARP14.2: n = 3; w/ 2-week IFNγ shNTC: n = 3; w/ 2-week IFNγ shPARP14.1: n = 3; w/ 2-week IFNγ shPARP14.2: n = 3) with or without chronic IFNγ treatment that expressed one of two PARP14-targeting shRNAs or a negative control shRNA (shNTC). GAPDH was used as a loading reference. The images were representatives of 1 of 3 independent experiments.

Gene	Fold Change (On/Pre)	P-value
PARP14	1.39	0.003
IFNG	1.81	<0.0001
STAT1	1.34	0.002

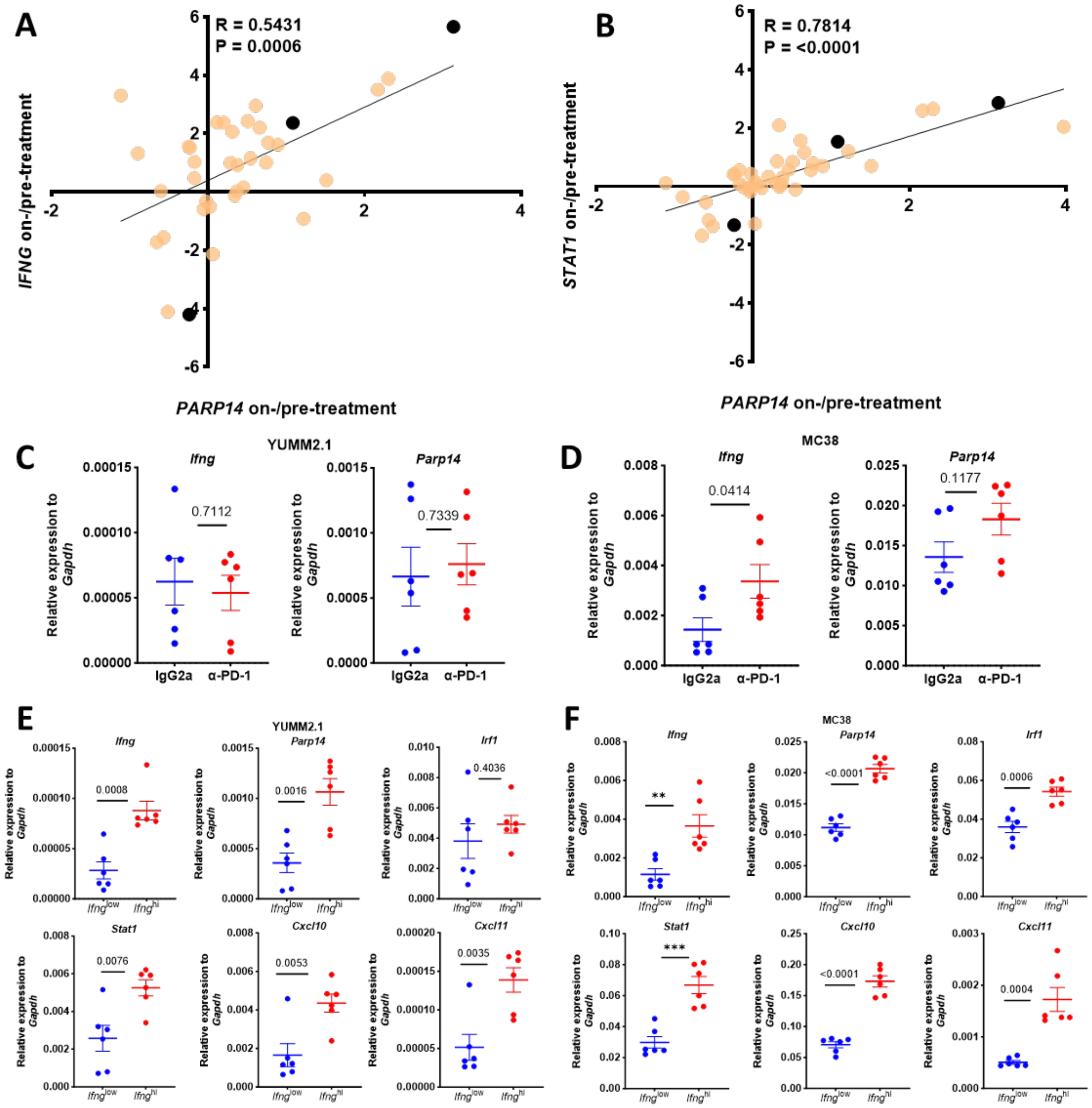

Supplementary Figure 12. *PARP14* mRNA expression is marginally upregulated in melanoma patients on α-PD-1 therapy while *Parp14* mRNA expression was higher in inflamed mouse tumours. (A–B) Average fold-change (table) and correlation of fold-change (scatter plots) of *PARP14* versus *IFNG* (A) and *STAT1* (B) mRNA in matching melanoma patient biopsies on-treatment versus pre-treatment with anti-PD-1 (n = 49, data retrieved from [2]). Spearman correlation co-efficient R and associated P-values were shown. Complete responders indicated as black dots. (C–F) 8–12-week-old wild-type C57BL/6 mice were subcutaneously implanted with IFNγ- naïve YUMM2.1 or MC38 cells. Treatment with α-PD-1 (YUMM2.1: n = 6; MC38: n = 6) or IgG2a antibodies (YUMM2.1: n = 6; MC38: n = 6) (two doses, three days apart) was initiated once tumour volume reached ~100–150 mm^3. Within 48-hour of the last dose being administered, tumours were dissected, and the RNA was extracted and subjected to RT-qPCR analysis. *Ifng* and *Parp14* mRNA expression in mice receiving α-PD-1 (n = 6) or IgG2a (n = 6). (C and E) YUMM2.1 and (D and F) MC38 αPD-1 or IgG2a-treated tumours were reclassified as $Ifng^{high}$ (n = 6) or $Ifng^{low}$ (n = 6) based on median expression levels. Expression levels for various STAT1 target genes (*Stat1*, *Irf1*, *Parp14*, *Cxcl10*, and *Cxcl11*) in these two cohorts were shown relative to the housekeeping gene *Gapdh*. The data were presented as mean ± S.E.M. and the p-values were determined by two-tailed unpaired t-test. Source data are provided as a Source Data file.

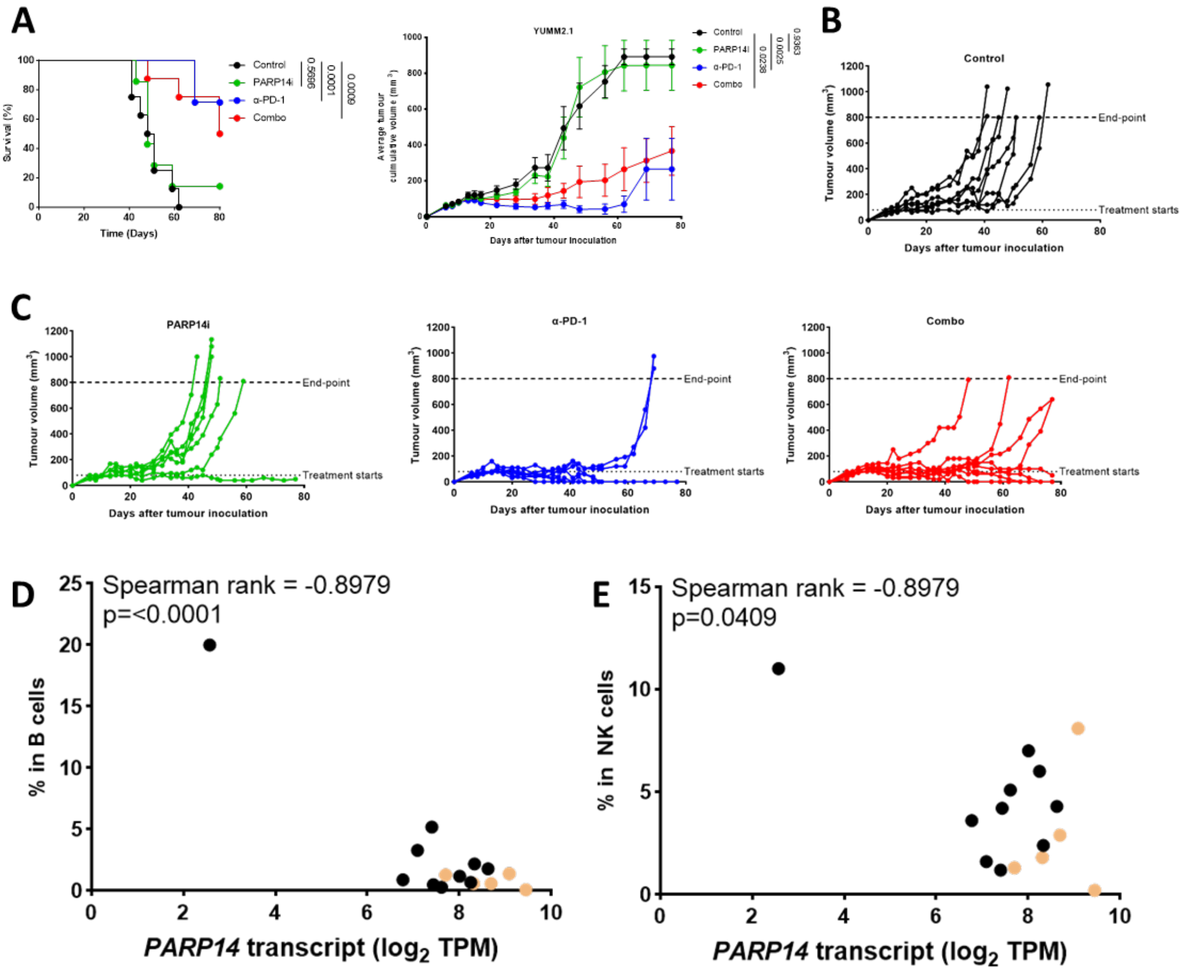

A
Survival (%)
Time (Days)
Control
PARP14i
α-PD-1
Combo
0.5696
0.0001
0.0009
YUMM2.1
Average tumour cumulative volume (mm³)
Days after tumour inoculation
0.0238
0.0025
0.9083
B
Control
Tumour volume (mm³)
End-point
Treatment starts
Days after tumour inoculation
C
PARP14i
α-PD-1
Combo
D
Spearman rank = -0.8979
p=<0.0001
% in B cells
PARP14 transcript (log2 TPM)
E
Spearman rank = -0.8979
p=0.0409
% in NK cells
PARP14 transcript (log2 TPM)

Supplementary Figure 13. PARP14 levels are augmented in tumours spontaneously relapsing after α-PD-1 treatment wherein it mediates resistance. (A–C) IFNγ-naïve YUMM2.1 cells were subcutaneously implanted into 8–12-week-old wild-type syngeneic female mice. Treatment with either α-PD-1 or IgG2a antibody was initiated once tumour volume reached 80-100 mm^3, with antibodies administered every three days for a total of four doses. In parallel, the animals also received two daily doses of the PARP14 inhibitor (PARP14i) RBN012759 or vehicle for a total of three weeks. (A) Kaplan-Meier plots (left) of IFNγ-naïve YUMM2.1 tumours in different treatment arms (Control: n = 8; PARP14i: n = 7; α-PD-1: n = 7; Combo: n = 8). The p-values were determined by Log-rank (Mantel-Cox) test. (Right) Average tumour growth curve for IFNγ-naïve YUMM2.1 cells after tumour inoculation in different treatment arms (Control: n = 8; PARP14i: n = 7; α-PD-1: n = 7; Combo: n = 8). The data were presented as mean ± S.E.M. and the adjusted p-values were determined by one-way ANOVA Tukey's test. (B–C) Individual tumour growth curve per treatment arm. (D–E) *PARP14* transcript abundance (log_2 TPM) scatter plots showing correlation with (D) B cells (n = 15) and (E) NK cells (n = 15) in the immune infiltration of the corresponding patients. Spearman correlations and P-values were shown. Patients with high intrinsic IFNγ signalling were labelled as orange dots. Source data are provided as a Source Data file.

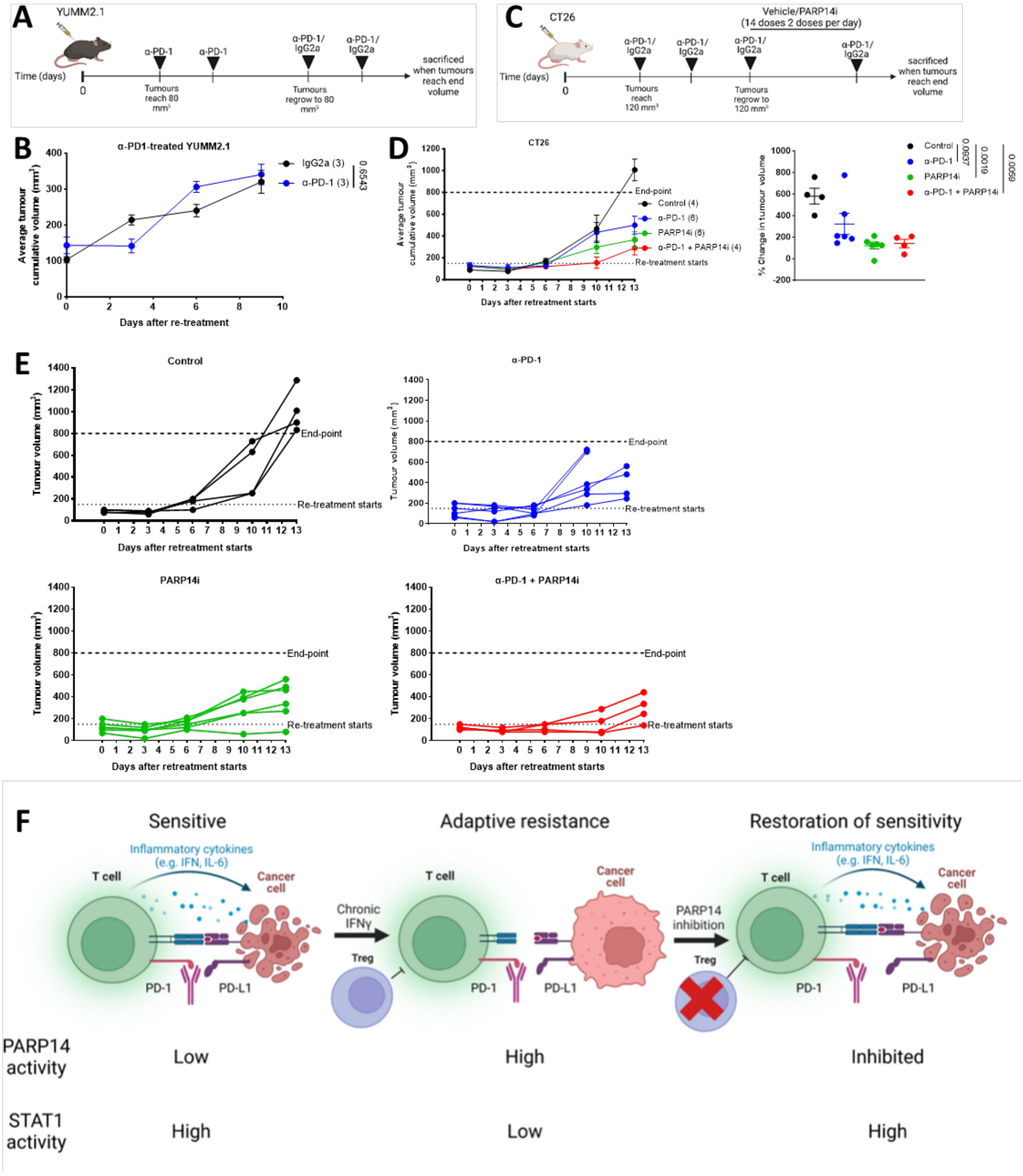
A
YUMM2.1
Time (days)
0
α-PD-1
α-PD-1
α-PD-1/ IgG2a
α-PD-1/ IgG2a
Tumours reach 80 mm³
Tumours regrow to 80 mm³
sacrificed when tumours reach end volume
C
CT26
Vehicle/PARP14i (14 doses 2 doses per day)
Time (days)
0
α-PD-1/ IgG2a
α-PD-1/ IgG2a
α-PD-1/ IgG2a
α-PD-1/ IgG2a
Tumours reach 120 mm³
Tumours regrow to 120 mm³
sacrificed when tumours reach end volume
B
α-PD1-treated YUMM2.1
Average tumour cumulative volume (mm³)
Days after re-treatment
IgG2a (3)
α-PD-1 (3)
0.6543
D
CT26
Average tumour cumulative volume (mm³)
Days after retreatment starts
End-point
Re-treatment starts
Control (4)
α-PD-1 (6)
PARP14i (6)
α-PD-1 + PARP14i (4)
% Change in tumour volume
Control
α-PD-1
PARP14i
α-PD-1 + PARP14i
0.0937
0.0019
0.0069
E
Control
α-PD-1
PARP14i
α-PD-1 + PARP14i
Tumour volume (mm³)
Days after retreatment starts
End-point
Re-treatment starts
F
Sensitive
Adaptive resistance
Restoration of sensitivity
Inflammatory cytokines (e.g. IFN, IL-6)
T cell
Cancer cell
PD-1
PD-L1
Chronic IFNγ
Treg
PARP14 inhibition
PARP14 activity
Low
High
Inhibited
STAT1 activity
High
Low
High

Supplementary Figure 14. Additional α-PD-1 doses failed to control YUMM2.1 tumour relapse. α-PD-1 and PARP14i combination therapy failed to synergise against CT26 relapsing tumours. (A) 8–12-week-old wild-type C57BL/6 mice were subcutaneously implanted with YUMM2.1 cells. Treatment with α-PD-1 antibodies (two doses, three days apart) was initiated once tumour volume reached ~80 mm^3. Once the tumours had regrown to roughly the size at which α-PD-1 treatment was initially commenced, they were treated with two doses of IgG2a (n = 3) or α-PD-1 (n = 3) (three days apart). (B) The average cumulative tumour volume growth curve was shown for each treatment condition. The data was presented as mean ± S.E.M. and the p-values were determined by two-tailed unpaired t test. (C) 8–12-week-old wild-type BALB/C mice were subcutaneously implanted with CT26 cells. α-PD-1 antibody treatment (two doses, three days apart) was initiated once tumour volume reached ~120 mm^3. Tumours regrew and upon reaching 120 mm^3, the tumours were treated with vehicle (n = 4), two doses of α-PD-1 antibodies (n = 6) (1 dose every 3 days), two daily doses of PARP14i (n = 6), or combination therapy (n = 4) for a week. (D) Graphs show average tumour cumulative volume (left) and percentage of tumour volume change between the start of α-PD-1 treatment and the end of re-treatment (right). The data was presented as mean ± S.E.M. and the adjusted p-values were assessed by one-way ANOVA Tukey's test. (E) Growth curves for individual tumours. (F) Proposed model of response to PD- 1 immune checkpoint inhibition restoration by PARP14 inhibition in tumours with resistance driven by IFNγ. Source data are provided as a Source Data file.

Supplementary Figure 15: Uncropped scans of all blots in supplementary figures.

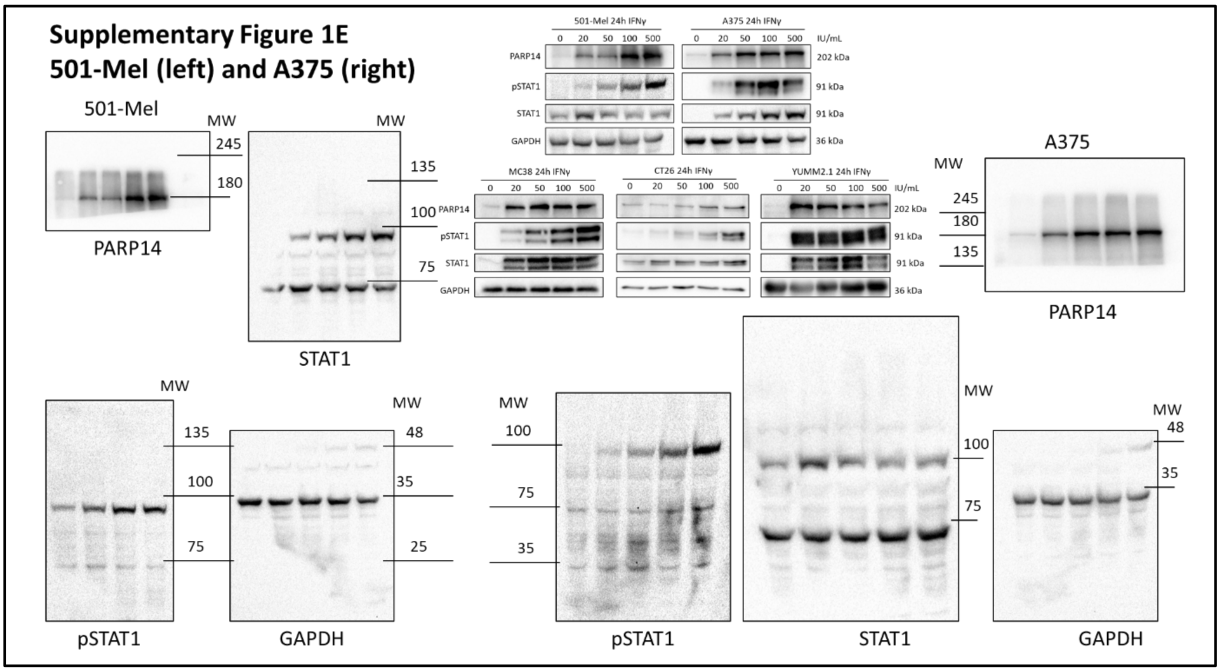

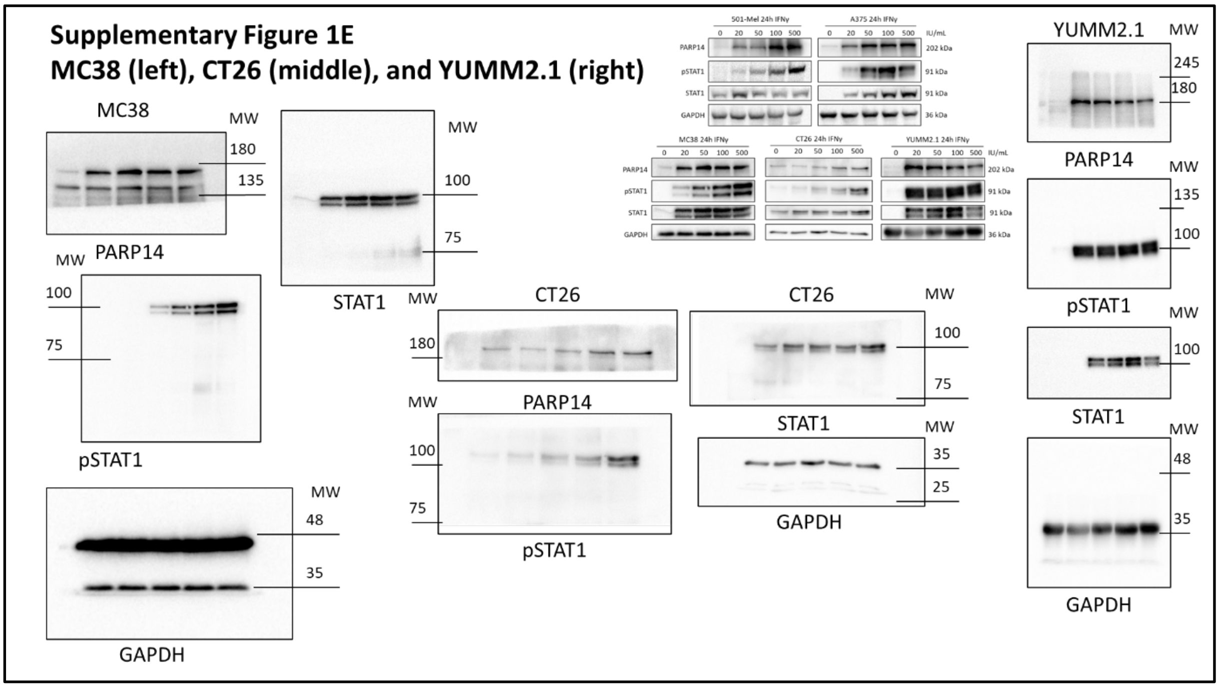

Supplementary Figure 1F
501-Mel

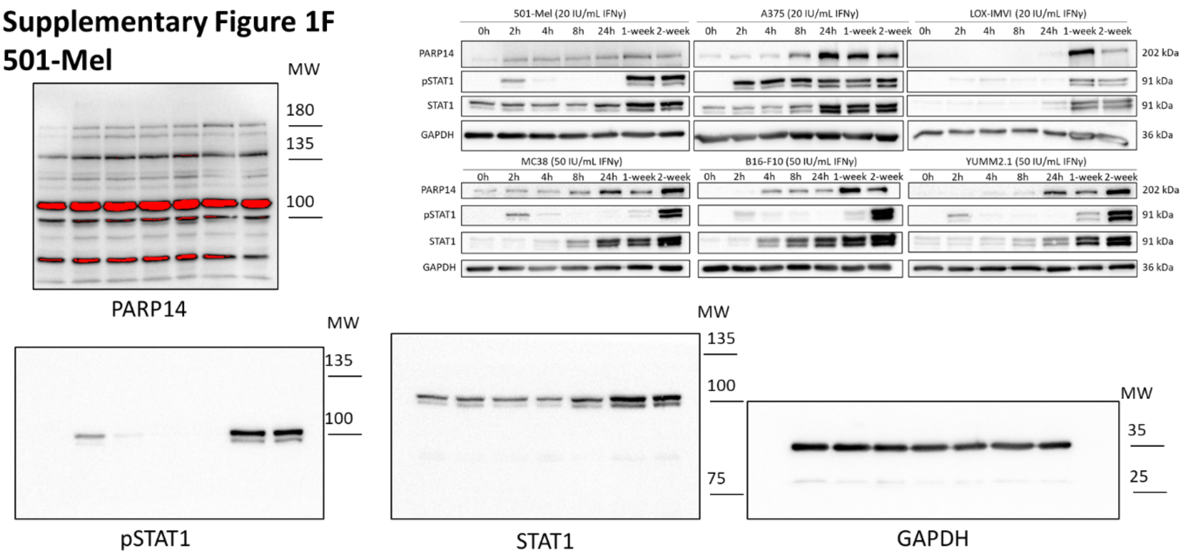

Supplementary Figure 1F
A375

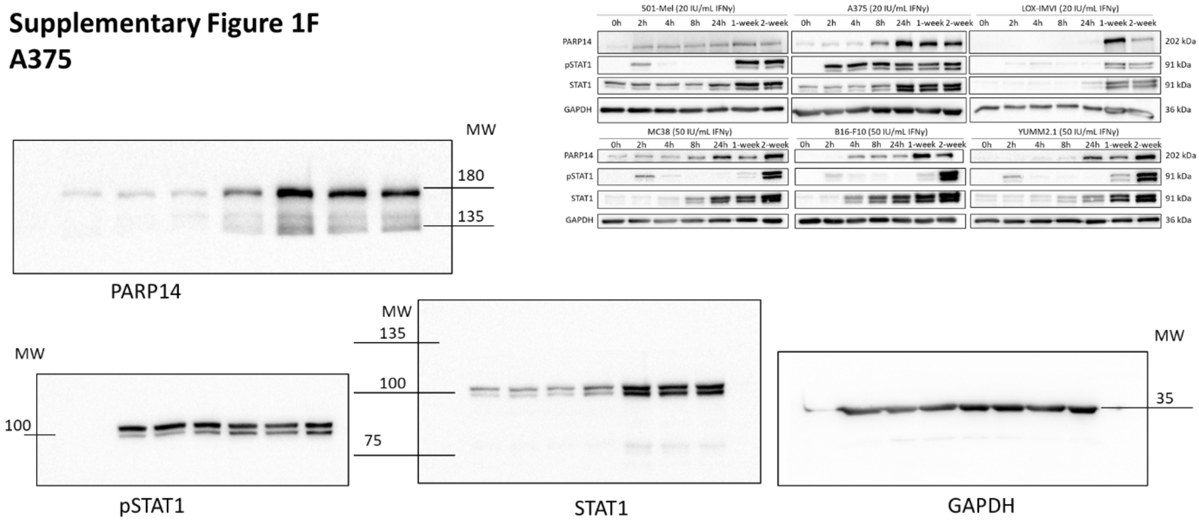

Supplementary Figure 1F
LOX-IMVI

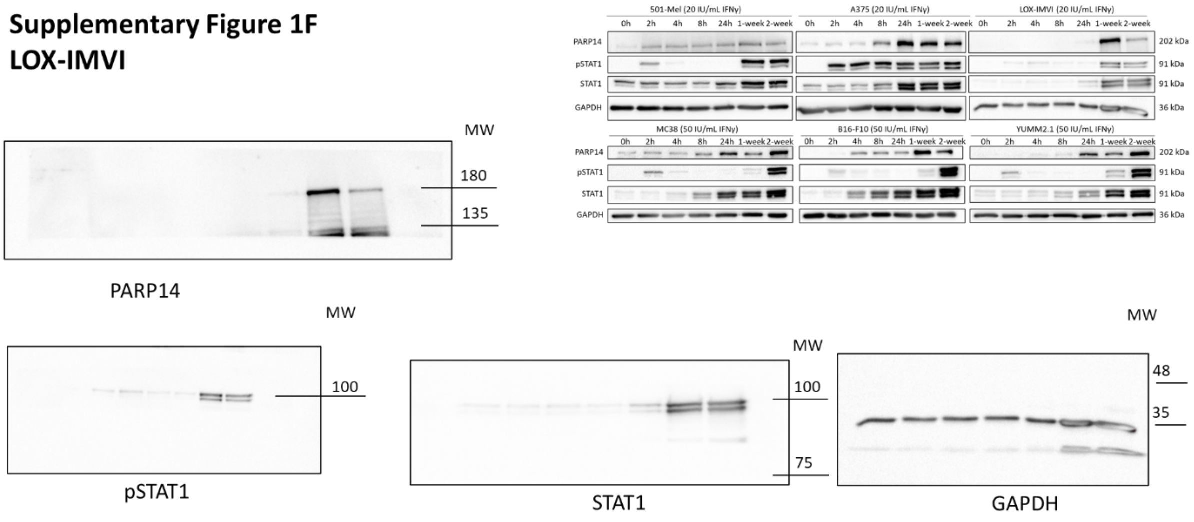

Supplementary Figure 1F
MC38

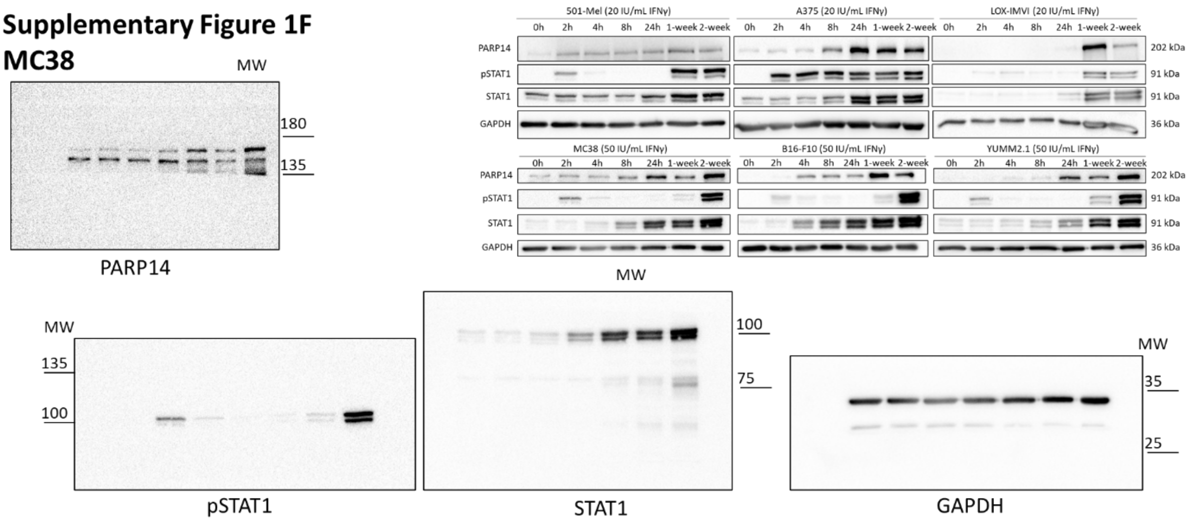

Supplementary Figure 1F
B16-F10

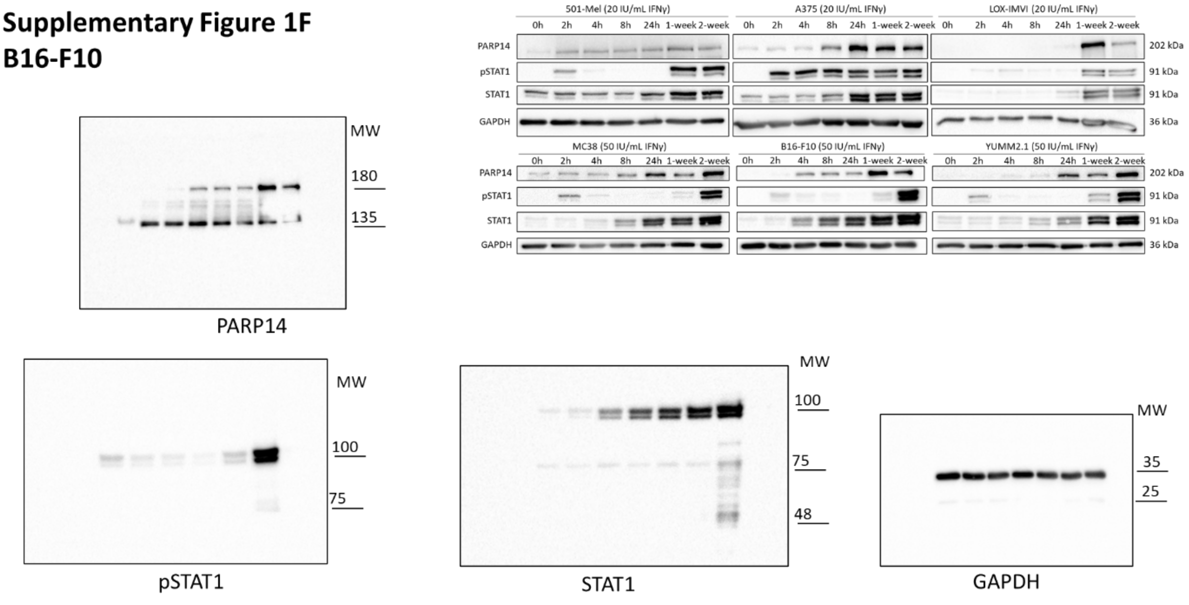

Supplementary Figure 1F
YUMM2.1

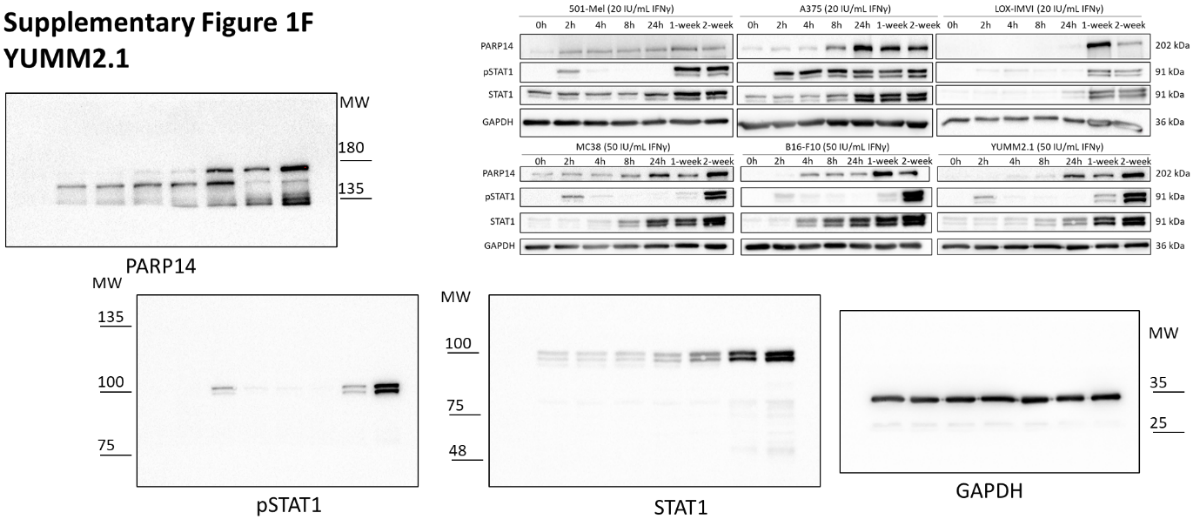

Supplementary Figure 11A
MV3

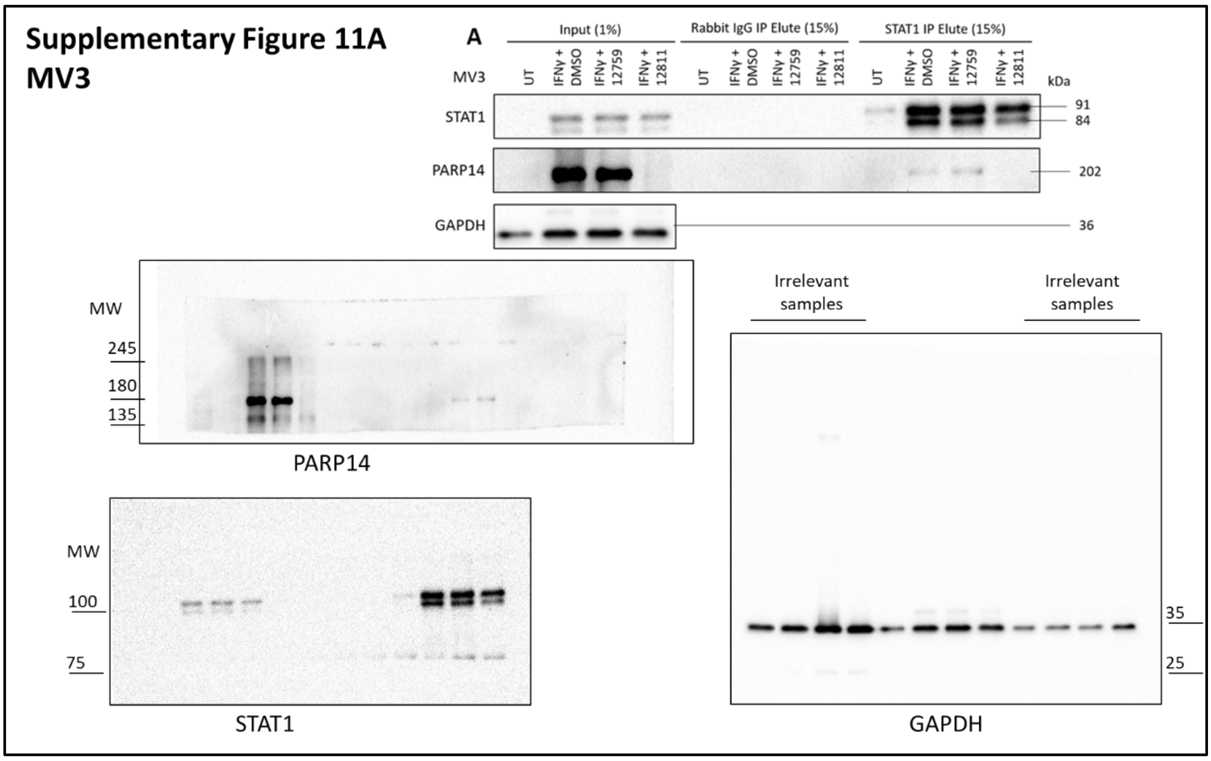

Supplementary Figure 11B
LOX-IMVI

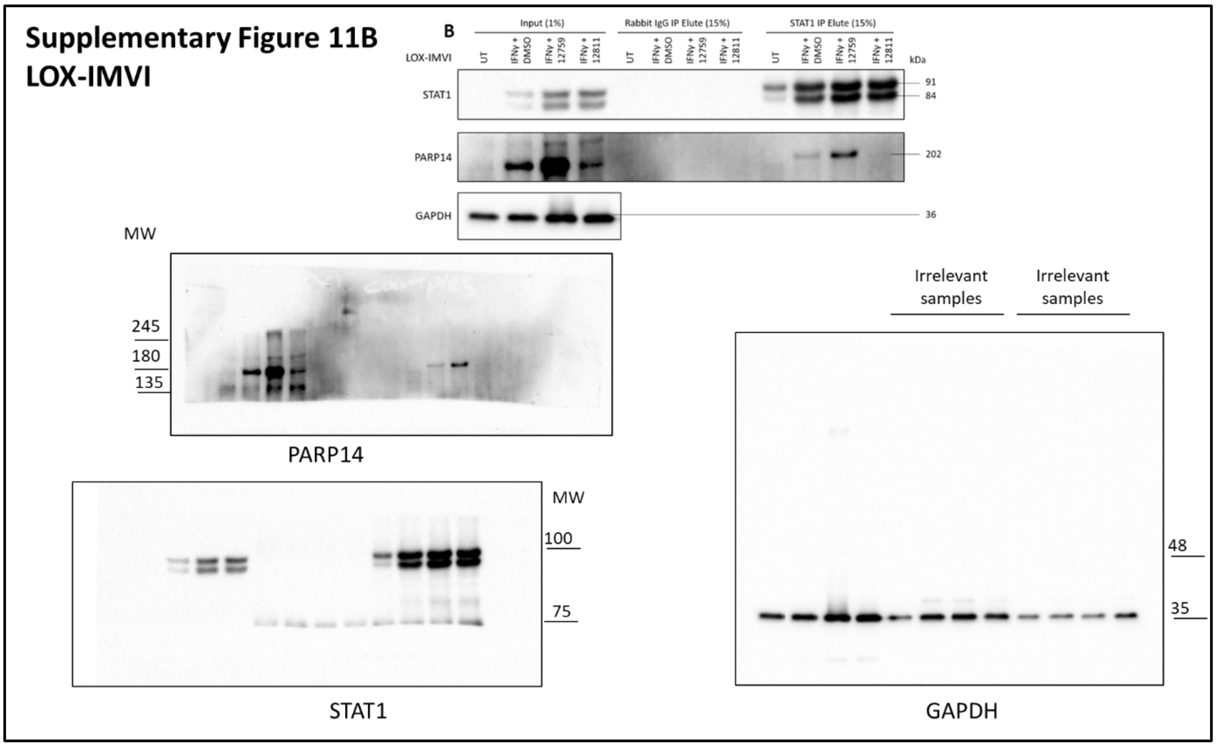

Supplementary Figure 11C
YUMM2.1

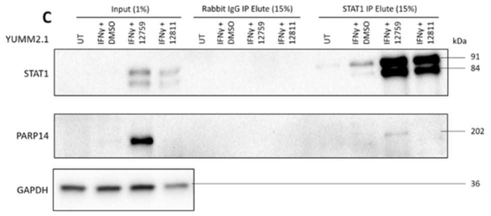

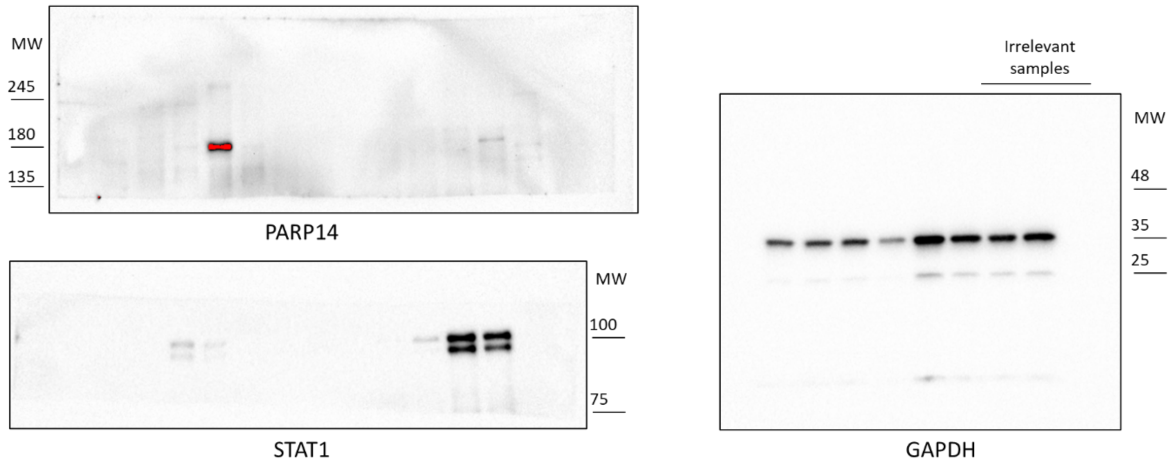

Supplementary Figure 11D
YUMM2.1 (left) and MC38 (right)

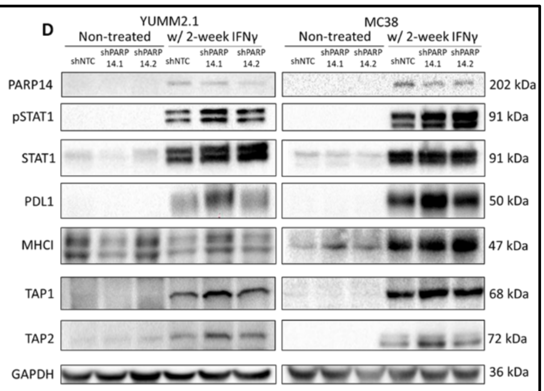

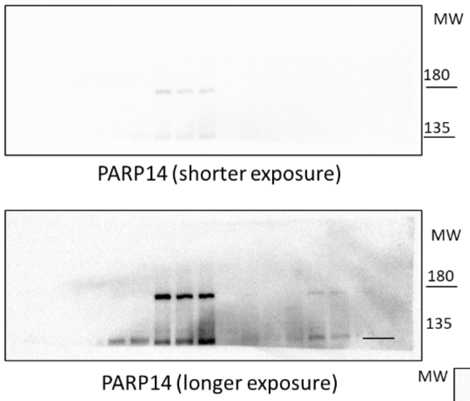

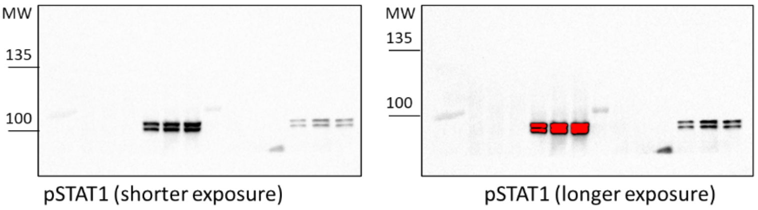

Supplementary Figure 11D
YUMM2.1 (left) and MC38 (right)

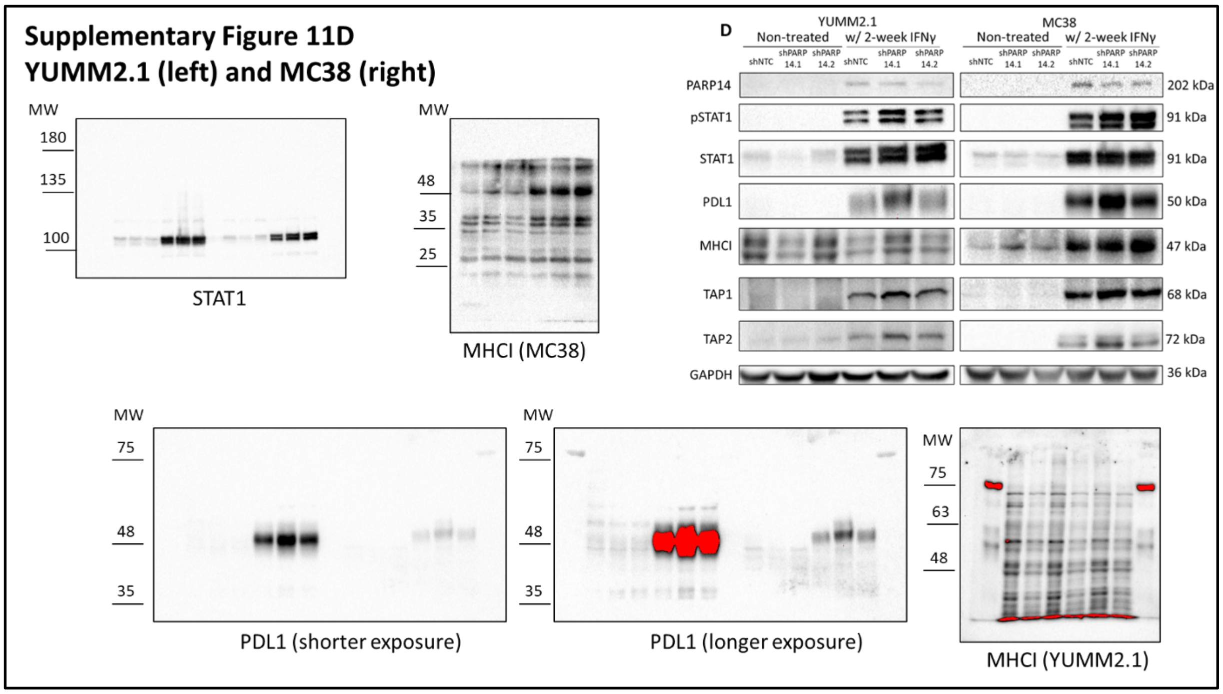

Supplementary Figure 11D
YUMM2.1 (left) and MC38 (right)

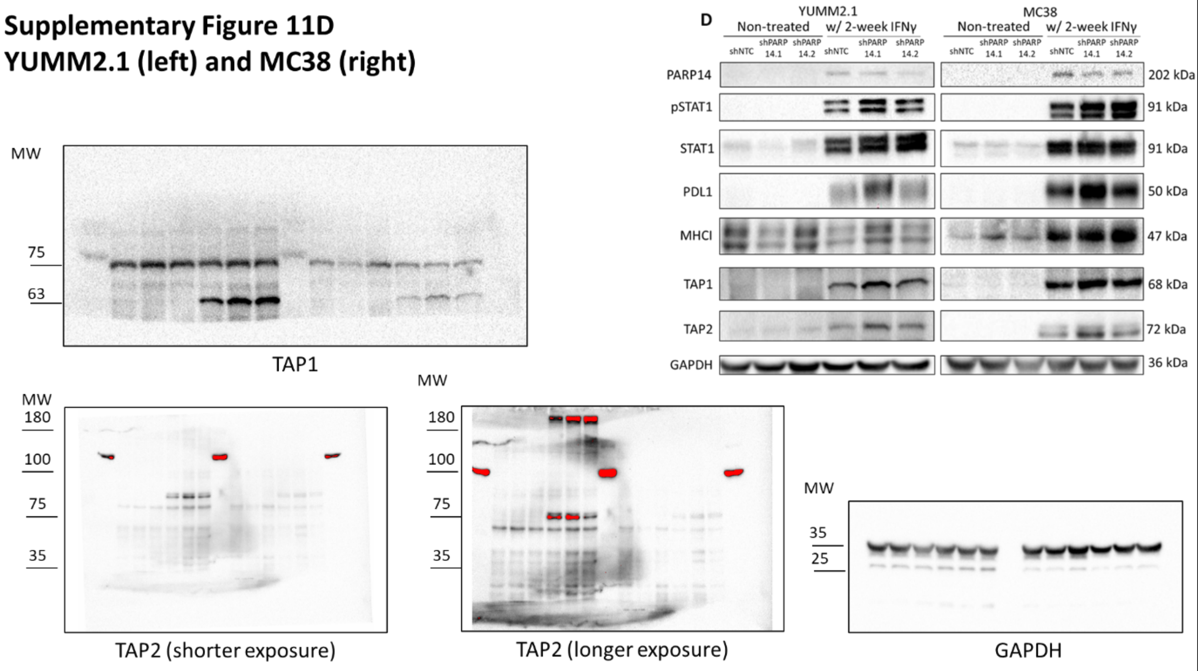

Supplementary Table 1. Oligonucleotides.

Gene	5’-3’ sequence
**RT qPCR Primers (Mouse)**	
Gapdh	Forward: TCTCCCTCACAATTTCCATCCCAG<br>Reverse: GGGTGCAGCGAACTTTATTGATGG
Ifng	Forward: ATGAACGCTACACACTGCATC<br>Reverse: CCATCCTTTTGCCAGTTCCTC
Parp14	Forward: CTATGCTGGGAAGAACGCTACT<br>Reverse: TGTAGTTTCCAGTGAGAACCCG
Irf1	Forward: TACCTGGGTCAGGACTTGGATA<br>Reverse: TCAGAGAGACTGCTGCTGAC
Stat1	Forward: GCCTCTCATTGTCACCGAAGAA<br>Reverse: TGGCTGACGTTGGAGATCACCA
Cxcl10	Purchased from Sino Biological, catalogue no. MP200169
Cxcl11	Purchased from Sino Biological, catalogue no. MP200181
Cd274	Purchased from Sino Biological, catalogue no. MP200010
Tnfa	Forward: CAACCTCCTCTCTGCCGTCAA<br>Reverse: CGTGGAAATTGGGTGTCCC
Tgfb	Forward: CGTGGAACTCTACCAGAAATACAGC<br>Reverse: TCAAAAGACAACCACTCAGGCG
Il10	Forward: TAAGGGTTACCTGGGTTGCCAAG<br>Reverse: CAAATGCTCCTTGATTTCTGGGC
**Short-hairpin (sh)RNAs**	
PARP14_Mouse_1 (shPARP14.1 in pLV-EGFP)	ACACCGGCTACGGGAAAGGAACCTATTTCTCGAGAAAT AGGTTCCTTTCCCGTAG<br>Purchased from VectorBuilder
PARP14_Mouse_2 (shPARP14.2 in pLV-EGFP)	ACACCGGAGAATGTGACCAGATAGTAAACTCGAGTTTA CTATCTGGTCACATTCT<br>Purchased from VectorBuilder
Non-target control shRNA (shNTC in pLV-EGFP)	CCTAAGGTTAAGTCGCCCTCG<br>Purchased from VectorBuilder
**Small interfering (si)RNAs**	
STAT1_Human_1	GCACGAUGGGCUCAGCUUU
STAT1_Human_2	CUACGAACAUGACCCUAUC
STAT1_Human_3	GAACCUGACUUCCAUGCGG
STAT1_Human_4	AGAAAGAGCUUGACAGUAA
Scramble	UGGUUUACAUGUCGACUAATT

1. Mariathasan S*, et al.* TGFβ attenuates tumour response to PD-L1 blockade by contributing to exclusion of T cells. *Nature* **554**, 544-548 (2018).

2. Riaz N*, et al.* Tumor and Microenvironment Evolution during Immunotherapy with Nivolumab. *Cell* **171**, 934-949.e916 (2017).
