# Supplementary material for: PARP14 inhibition restores PD-1 immune checkpoint inhibitor response following IFNγ-driven acquired resistance in preclinical cancer models
Source: Nat Commun. 2023 Sep 26;14:5983. doi: 10.1038/s41467-023-41737-1 (PMC10522711; doi:10.1038/s41467-023-41737-1)
Supplement: Supplementary file 3 — Reporting Summary [file 41467_2023_41737_MOESM3_ESM.pdf]

nature portfolio

Corresponding author(s): Adam Hurlstone

Last updated by author(s): Sep 5, 2023

Reporting Summary

Nature Portfolio wishes to improve the reproducibility of the work that we publish. This form provides structure for consistency and transparency in reporting. For further information on Nature Portfolio policies, see our Editorial Policies and the Editorial Policy Checklist.

Statistics

For all statistical analyses, confirm that the following items are present in the figure legend, table legend, main text, or Methods section.

n/a	Confirmed	
☐	☒	The exact sample size (*n*) for each experimental group/condition, given as a discrete number and unit of measurement
☐	☒	A statement on whether measurements were taken from distinct samples or whether the same sample was measured repeatedly
☐	☒	The statistical test(s) used AND whether they are one- or two-sided<br>*Only common tests should be described solely by name; describe more complex techniques in the Methods section.*
☐	☒	A description of all covariates tested
☐	☒	A description of any assumptions or corrections, such as tests of normality and adjustment for multiple comparisons
☐	☒	A full description of the statistical parameters including central tendency (e.g. means) or other basic estimates (e.g. regression coefficient) AND variation (e.g. standard deviation) or associated estimates of uncertainty (e.g. confidence intervals)
☐	☒	For null hypothesis testing, the test statistic (e.g. *F*, *t*, *r*) with confidence intervals, effect sizes, degrees of freedom and *P* value noted<br>*Give P values as exact values whenever suitable.*
☒	☐	For Bayesian analysis, information on the choice of priors and Markov chain Monte Carlo settings
☒	☐	For hierarchical and complex designs, identification of the appropriate level for tests and full reporting of outcomes
☒	☐	Estimates of effect sizes (e.g. Cohen's *d*, Pearson's *r*), indicating how they were calculated

Our web collection on statistics for biologists contains articles on many of the points above.

Software and code

Policy information about availability of computer code

Data collection	MxPro qPCR software version 3.00<br>Image Lab™ Software (version 3.0.1.)<br>Hiseq Software Suite (version 3.4.0)<br>BD FACSDiva™ software<br>Biotek Synergy™ H1 Hybrid Multi-Mode Reader
Data analysis	GraphPad Prism (version 9.0)<br>MxPro qPCR software (version 3.00)<br>htseq-count (version 0.9.1)<br>edgeR (version 3.24.1)<br>Multiple Experiment Viewer (version 10.2)<br>tximport (v1.28.0)<br>trimmomatic (version 0.36.6; sliding window trimming with 4 bases averaging and average quality minimum set to 20)<br>HISAT2 (version 2.1.0; default parameters)<br>DEseq2<br>BubbleGUM software (version 1.3.19)<br>Broad Institute Molecular Signatures Database (MSigDB) (version 7.0)<br>Ingenuity Pathway Analysis (QIAGEN; version 01-12)<br>FlowJo (version 8.7)

ImmuneCellAI

For manuscripts utilizing custom algorithms or software that are central to the research but not yet described in published literature, software must be made available to editors and reviewers. We strongly encourage code deposition in a community repository (e.g. GitHub). See the Nature Portfolio guidelines for submitting code & software for further information.

Data

Policy information about availability of data

All manuscripts must include a data availability statement. This statement should provide the following information, where applicable:

- Accession codes, unique identifiers, or web links for publicly available datasets
- A description of any restrictions on data availability
- For clinical datasets or third party data, please ensure that the statement adheres to our policy

The RNA-seq data generated in this study have been deposited with ArrayExpress (https://www.ebi.ac.uk/biostudies/arrayexpress) Gene Expression Omnibus (GEO) (https://www.ncbi.nlm.nih.gov/geo/) with accession numbers EMTAB-12194, E-MTAB-12195, , E-MTAB-12872, E-MTAB-12196, and GSE237098. The TCGA data used are publicly available at the Genomic Data Commons portal (https://portal.gdc.cancer.gov/), where skin cutaneous melanoma (SKCM) patients data was used. Transcriptomic data—fragments per kilobase of transcript per million mapped fragments (FPKM) and transcripts per million (TPM) data— from pre-treatment and on-treatment biopsies of melanoma patients undergoing ICBT were obtained from the GEO database (https://www.ncbi.nlm.nih.gov/geo/) with accession number GSE91061; also from melanoma cell cultures from ICBT-progressing lesions in the Sequence Read Archive (https://www.ncbi.nlm.nih.gov/sra) under accession code PRJNA818797. TPM values were converted to log2(TPM+1). ChIP-seq data for STAT1 were retrieved from the Encyclopedia of DNA Elements (ENCODE) project database [https://www.encodeproject.org]. All the raw data is available through source data file.

Research involving human participants, their data, or biological material

Policy information about studies with human participants or human data. See also policy information about sex, gender (identity/presentation), and sexual orientation and race, ethnicity and racism.

Reporting on sex and gender	N/A
Reporting on race, ethnicity, or other socially relevant groupings	N/A
Population characteristics	N/A
Recruitment	N/A
Ethics oversight	N/A

Note that full information on the approval of the study protocol must also be provided in the manuscript.

Field-specific reporting

Please select the one below that is the best fit for your research. If you are not sure, read the appropriate sections before making your selection.

[x] Life sciences [ ] Behavioural & social sciences [ ] Ecological, evolutionary & environmental sciences

For a reference copy of the document with all sections, see nature.com/documents/nr-reporting-summary-flat.pdf

Life sciences study design

All studies must disclose on these points even when the disclosure is negative.

Sample size	A minimum of 3 for in vivo and minimum of 3 in vitro independent replicates were used for all samples unless otherwise specified in the manuscript. This sample size was determined to be sufficient for calculating average and standard error and eventually statistical significance amongst different groups. Sample sizes were chosen by performing power analysis with an alpha of 0.05 and a power of 80%.
Data exclusions	No data were excluded from analysis.
Replication	All experimental results and conclusions were derived from using independent biological replicates.
Randomization	Mice procured from the same source/vendor were randomly assigned to a treatment group. Mice were housed in random cages before experiments for in vivo studies. Sex was not considered. In vitro experiments of cells was done randomly (At least 3 independent replicates).
Blinding	For in vivo studies, tumour inoculation, treatments, tumour measurement and analysis was conducted by different investigators and the investigator providing the treatment was not blinded since this is not possible, however, in each of the subsequent steps the scientists were not aware of the treatment groups.

Reporting for specific materials, systems and methods

We require information from authors about some types of materials, experimental systems and methods used in many studies. Here, indicate whether each material, system or method listed is relevant to your study. If you are not sure if a list item applies to your research, read the appropriate section before selecting a response.

Materials & experimental systems

n/a	Involved in the study
☐	☒ Antibodies
☐	☒ Eukaryotic cell lines
☒	☐ Palaeontology and archaeology
☐	☒ Animals and other organisms
☒	☐ Clinical data
☒	☐ Dual use research of concern
☒	☐ Plants

Methods

n/a	Involved in the study
☒	☐ ChIP-seq
☐	☒ Flow cytometry
☒	☐ MRI-based neuroimaging

Antibodies

Antibodies used

Antibodies for western blot:
Target Company Catalog # Dilution
PARP14 C-1 Santa Cruz Biotechnology sc-377150 1:500
Phospho-Stat1 (Tyr701) 58D6 Cell Signalling Technology 9167L 1:1000
STAT1 Cell Signalling Technology 9172 1:1000
MHC Class I H2 Kb Abcam ab93364 1:500
GAPDH Proteintech 60004-1-Ig 1:20000
TAP1 Cell Signalling Technology 12341 1:1000
Human/ Mouse TAP2 Cell Signalling Technology 12259 1:1000; Santa Cruz Biotechnology, sc-515576 1:500
Mouse/ Human PD-L1/B7-H1 R&D Systems AF1019-SP 1:500 Cell Signalling Technology, 13684 1:1000
Anti-mouse IgG HRP Cell Signaling Technology 7076S 1:5000
Anti-rabbit IgG HRP GE Healthcare NA934 1:5000
Anti-goat IgG HRP Santa Cruz Biotechnology sc-2020 1:5000

Antibodies for flow cytometry:
Target Fluorophore Company Catalog # Clone Dilution
CD3 BioLegend 100238 (clone 17A2) 3 mg/mL
CD28 BioLegend 102132 (clone 37.51) 3 mg/mL
CD16/32 BioLegend 101301 (clone 93) 1:50
CD45 PerCP-Cy5.5 BioLegend 103131 (clone 30-F11) 1:100
CD45 BV510 BioLegend 103137 (clone 30-F11) 1:20
CD3 VioBright FITC BioLegend 100306 (clone 145-2C11) 1:50
CD4 APC Vio770 BioLegend 100414 (clone GK1.5) 1:20
CD4 AF700 BioLegend 100536 (clone GK1.5) 1:100
CD8α BV650 BioLegend 100741 (clone 53-6.7) 1:100
CD8α PE-Cy7 BioLegend 100721 (clone 53-6.7) 1:80
CD8α PerCP-Cy5.5 BioLegend 100734 (clone 53-6.7) 1:20
CD8α BV711 BioLegend 100759 (clone 53-6.7) 1:40
CD25 BV650 BioLegend 102037 (clone PC61) 1:20
PD-1 V710/50 BioLegend 135231 (clone 29F.1A12) 1:100
Granzyme B PE BioLegend 372208 (clone QA16A02) 1;20
LAG-3 PE-CF594 BioLegend 125224 (clone C9B7W) 1:40
TIM-3 PE-Cy7 BioLegend 119716 (clone RMT3-23) 1:40
TCR γ/δ PE BioLegend 118107 (clone GL3) 1:200
NK1.1 PE-CF594 BioLegend 108715 (clone PK136) 1:50
IFNγ V450 BioLegend 505829 (clone XMG1.2) 1:20
TNF-α BV786 BioLegend 506341 (clone MP6-XT22) 1:80
LAP PE BioLegend 141305 (clone TW7-20B9) 1:40
IL-10 PE-CF594 BioLegend 505033 (clone JES5-16E3) 1:80
Ki-67 BV650 BioLegend 151215 (clone 11F6) 1:100
FOXP3 V450 eBioscience 48577382 (clone FJK-16s) 1:100
TCR beta APC-Vio770 eBioscience 47-596182 (clone H57-597) 1:20

Antibodies for in vivo studies:
Target Company Catalog # Clone Amount per dose
IgG2a BioXCell BE0089 2A3 300 µg
IgG2b BioXCell BE0090 LTF-2 100 µg
PD-1 BioXCell BE0146 RMP1-14 300 µg
CD8α BioXCell BE0061 2.43 100 µg

Validation

All antibodies were validated by the manufacturer, and antibody-specific staining was compared to fluorescent minus one control samples in flow cytometry or isotype antibody in vivo studies.

For primary antibodies used in western blot:
PARP14 C-1 Santa Cruz Biotechnology sc-377150 1:500: According to web site (https://www.scbt.com/p/parp-14-antibody-c-1), the antibody is validated at 1:500 dilution for WB in multiple studies (e.g. PMID: 36030235).
Phospho-Stat1 (Tyr701) 58D6 Cell Signalling Technology 9167L 1:1000: According to web site (https://www.cellsignal.com/products/primary-antibodies/phospho-stat1-tyr701-58d6-rabbit-mab/9167), the antibody is validated at 1:1000 dilution for WB in multiple studies (e.g. PMID: 37454216).
STAT1 Cell Signalling Technology 9172 1:1000: According to web site (https://www.cellsignal.com/products/primary-antibodies/stat1-antibody/9172), the antibody is validated at 1:1000 dilution for WB in multiple studies (e.g. PMID: 37454216).
MHC Class I H2 Kb Abcam ab93364 1:500: According to web site (https://www.citeab.com/antibodies/738999-ab93364-anti-mhc-class-i-h2-kb-antibody), the antibody is validated at 1:500 dilution for WB in multiple studies (e.g. PMID: 36849929).
GAPDH Proteintech 60004-1-Ig 1:20000: According to web site (https://www.ptglab.com/products/GAPDH-Antibody-60004-1-Ig.htm), various lysates (HeLa, HEK-293, HepG2, HSC-T6) were subjected to SDS PAGE followed by western blot with 60004-1-Ig (GAPDH antibody) at dilution of 1:50000 incubated at room temperature for 1.5 hours as the validation.
TAP1 Cell Signalling Technology 12341 1:1000: According to web site (https://www.cellsignal.com/products/primary-antibodies/tap1-antibody/12341), the antibody is validated at 1:1000 dilution for WB in multiple studies (e.g. PMID: 35775490).
Human/ Mouse TAP2 Cell Signalling Technology 12259 1:500; Santa Cruz Biotechnology, sc-515576 1:500: According to web site (https://www.cellsignal.com/products/primary-antibodies/tap2-antibody/12259 and https://www.scbt.com/p/tap2-antibody-b-2), the antibody is validated at 1:1000 and 1:500 dilution for WB in multiple studies (e.g. PMID: 35775490 and PMID: 36430709).
Mouse/ Human PD-L1/B7-H1 R&D Systems AF1019-SP 1:500 Cell Signalling Technology, 13684 1:1000: According to web site (https://www.rndsystems.com/products/mouse-pd-l1-b7-h1-antibody_af1019 and https://www.cellsignal.com/products/primary-antibodies/pd-l1-e1l3n-xp-rabbit-mab/13684), the antibody is validated at 1:500 using RAW 264.7 mouse monocyte/macrophage cell line untreated (-) or treated (+) with 10 µg/mL LPS for 4 hours and 1:1000 dilution for WB for multiple studies (e.g. PMID: 37340461).
Anti-mouse IgG HRP Cell Signaling Technology 7076S 1:5000: According to web site (https://www.cellsignal.com/products/secondary-antibodies/anti-mouse-igg-hrp-linked-antibody/7076), the antibody is validated at 1:5000 dilution for WB in multiple studies (e.g. PMID: 36633363).
Anti-rabbit IgG HRP GE Healthcare NA934 1:5000: According to web site (https://www.sigmaaldrich.com/GB/en/product/sigma/gena9341ml), the antibody is validated at 1:5000 dilution for WB in multiple studies (e.g. PMID: 33857306).
Anti-goat IgG HRP Santa Cruz Biotechnology sc-2020 1:5000: According to web site (chrome-extension://efaidnbmnnnibpcajpcglclefindmkaj/https://datasheets.scbt.com/sc-2020.pdf), the antibody is validated at 1:5000 dilution for WB in multiple studies (e.g. PMID: 9765152).

For flow cytometry:
CD3: Induction of a cellular active state from a normal resting state, C57BL/6 splenocytes; Manufacturer
CD28: Induction of a cellular active state from a normal resting state, C57BL/6 splenocytes; Manufacturer
CD3 PerCP/Cyanine5.5 anti-mouse; C57BL/6 mouse splenocytes stained with purified 93, followed by anti-rat IgGs FITC; Manufacturer
CD45 PerCP-Cy5.5 anti-mouse; C57BL/6 mouse splenocytes were stained with CD45 (Clone 30-F11) PerCP/Cyanine5.5 (filled histogram), or rat IgG2b, PerCP/Cyanine5.5 isotype control; Manufacturer
CD45 BV510 anti-mouse; C57BL/6 mouse splenocytes were stained with CD45 (clone 30-F11) Brilliant Violet 510™; Manufacturer
CD3 anti-mouse; C57BL/6 mouse splenocytes were stained with CD3e (clone 145-2C11) FITC and CD127 (clone SB/199) PE; Manufacturer
CD4 anti-mouse; C57BL/6 mouse splenocytes were stained with CD3 FITC and CD4 (clone GK1.5) APC/Cyanine7 (left) or Rat IgG2b, APC/Cyanine7 isotype control (right); Manufacturer
CD4 anti-mouse; C57BL/6 mouse splenocytes stained with CD4 (clone RM4-5) Alexa Fluor® 700 (filled histogram) or rat IgG2a, κ Alexa Fluor® 700 isotype control (open histogram); Manufacturer
CD8α anti-mouse; C57BL/6 mouse splenocytes were stained with CD3 PE and CD8a (clone 53-6.7) Brilliant Violet 650™; Manufacturer
CD8α anti-mouse; C57BL/6 splenocytes were stained with CD8 (clone 53-6.7) PE/Cyanine7 and CD3 FITC; Manufacturer
CD8α anti-mouse; C57BL/6 mouse splenocytes were stained with CD3ε FITC and CD8a (clone 53-6.7) PerCP/Cyanine5.5, or Rat IgG2a, κ PerCP/Cyanine5.5 isotype control (bottom); Manufacturer
CD8α anti-mouse; C57BL/6 mouse splenocytes were stained with CD3 PE and CD8a (clone 53-6.7) Brilliant Violet 711™; Manufacturer
CD25 anti-mouse; C57BL/6 mouse splenocytes were stained with CD4 FITC and CD25 (clone PC61) Brilliant Violet 650™; Manufacturer
PD-1 anti-mouse; Con-A and IL-2 stimulated C57BL/6 splenocytes (three days) were stained with CD3 FITC and CD279 (clone 29F.1A12) Brilliant Violet 711™ (top), or rat IgG2a, κ Brilliant Violet 711™ isotype control (bottom); Manufacturer
Granzyme B anti-mouse; Human peripheral blood mononuclear cells were stained with CD8 Pacific Blue™, fixed and permeabilized, and then stained with Granzyme B PE (clone QA16A02, top) or mouse IgG1, κ PE isotype control (bottom); Manufacturer
LAG-3 anti-mouse; Con A+IL-2-stimulated C57BL/6 splenocytes (3 days) were stained with CD223 (clone C9B7W) PE/Dazzle™ 594 (filled histogram) or rat IgG1, κ PE/Dazzle™ 594 isotype control (open histogram); Manufacturer
TIM-3 anti-mouse; Mouse Tim-3 transfected cells were stained with anti-mouse CD366 (Tim-3, clone RMT3-23) PE/Cyanine7 (filled histogram) or rat IgG2a, κ PE/Cyanine7 isotype control (open histogram); Manufacturer
TCR γ/δ anti-mouse; C57BL/6 splenocytes stained with CD3 (145-2C11) APC and GL3 PE; Manufacturer
NK1.1 anti-mouse; C57BL/6 mouse splenocytes were stained with DX5 APC and NK1.1 (clone PK136) PE/Cyanine5; Manufacturer
IFNγ anti-mouse; C57BL/6 mouse splenocytes were stimulated with PMA + Ionomycin for 6 hours (in the presence of monensin), stained with CD3 FITC, fixed, permeabilized, and then stained with IFN-γ (clone XMG1.2) Brilliant Violet 421™ (top) or rat IgG1, κ Brilliant Violet 421™ isotype control (bottom); Manufacturer
TNF-α anti-mouse; PMA + Ionomycin-stimulated C57BL/6 mouse splenocytes (six-hour in the presence of monensin) were stained with CD3 APC, fixed, permeabilized and then stained with TNF-α (clone MP6-XT22) Brilliant Violet 785™ (top) or rat IgG1, κ Brilliant Violet 785™ isotype control (bottom); Manufacturer

LAP anti-mouse; C57BL/6 mouse splenocytes were stimulated with anti-mouse CD3, CD28, and recombinant mouse IL-2 for 48-hours, then surface stained with CD4 FITC and LAP (TGF-β1) (clone TW7-20B9) PE (top) or mouse IgG1, κ PE isotype control (bottom). This was followed by intracellular staining with FOXP3 Alexa Fluor® 647. Data shown was generated by gating on CD4+ lymphocyte population; Manufacturer
IL-10 anti-mouse; PMA+ionomycin-stimulated (six hours in the presence of monensin) Th2-polarized BALB/c splenocytes were stained with CD3 Alexa Fluor® 488, fixed, permeabilized, and then stained with IL-10 (clone JES5-16E3) PE/Dazzle™ 594 (top) or rat IgG2b, κ PE/Dazzle™ 594 isotype control (bottom); Manufacturer
Ki-67 anti-mouse; C57BL/6J splenocytes treated with ConA and IL-2 for 2 days were fixed, permeabilized with 70% ethanol, and stained with Ki-67 (clone 11F6) Brilliant Violet 650™ (filled histogram) or rat IgG2b, κ (clone RTK4530) Brilliant Violet™ 650 isotype control (open histogram); Manufacturer
FOXP3 anti-mouse; Surface staining of C57BL/6 splenocytes with Anti-Mouse CD4 FITC (Product # 11-0041-82) followed by fixation and permeabilization with the Foxp3 Staining Buffers (Product # 00-5523-00) and intracellular staining with 0.03 µg of Rat IgG2a kappa Isotype Control eFluor® 450 (Product # 48-4321-82) (left) or 0.03 µg of Anti-Mouse/Rat Foxp3 eFluor® 450 (right). Cells in the lymphocyte gate were used for analysis; Manufacturer
TCR beta anti-mouse; Staining of C57BL/6 splenocytes with Anti-Human/Mouse CD45R (B220) FITC (Product # 11-0452-82) and 0.5 µg of Armenian Hamster IgG Isotype Control APC-eFluor® 780 (Product # 47-4888-80) (left) or 0.5 µg of Anti-Mouse TCR beta APC-eFluor® 780 (right). Total viable cells were used for analysis; Manufacturer

For in vivo studies:
IgG2a: According to web site (https://bioxcell.com/invivomab-rat-igg2a-isotype-control-anti-trinitrophenol-be0089#tab_specifications), the antibody is validated for in vivo studies in multiple studies (e.g. PMID: 30097293).
IgG2b: According to web site (https://bioxcell.com/invivomab-rat-igg2b-isotype-control-anti-keyhole-limpet-hemocyanin-be0090), the antibody is validated for in vivo studies in multiple studies (e.g. PMID: 30097293).
PD-1: According to web site (https://bioxcell.com/invivomab-anti-mouse-pd-1-cd279-be0146), the antibody is validated for in vivo studies in multiple studies (e.g. PMID: 30010674).
CD8α: According to web site (https://bioxcell.com/invivomab-anti-mouse-cd8a-be0061?gad=1&gclid=CjwKCAjwo9unBhBTEiwAipC11ywLcn3Npyjt7VgL2RKM1MDjMrO2f5cpd-SvRk59ii8oebJ-UCG8uxoCdnUQAvD_BwE), the antibody is validated for in vivo studies in multiple studies (e.g. PMID: 29864117).

Eukaryotic cell lines

Policy information about cell lines and Sex and Gender in Research

Cell line source(s)	The human melanoma cell lines A375, LOX-IMVI and 501-Mel were generously gifted by Claudia Wellbrock, The University of Manchester, Lenti-X 293T cells were generously gifted Angeliki Malliri, The University of Manchester, 5555, B16-F10, and MC38 cells were generously giftedby Santiago Zelenay, The University of Manchester and YUMM2.1 cells were generously gifted by Richard Marais, The University of Manchester.
Authentication	Cancer cell lines have been authenticated using STR profiling, except for MC38 for which no reference data set are available to compare.
Mycoplasma contamination	Cell cultures were routinely tested for mycoplasma contamination by PCR and deemed to be uninfected.
Commonly misidentified lines (See ICLAC register)	No commonly misidentified cell lines were used in the study.

Animals and other research organisms

Policy information about studies involving animals; ARRIVE guidelines recommended for reporting animal research, and Sex and Gender in Research

Laboratory animals	Female 6-8 week-old C57BL/6J and BALB/C mice were used for the entire study. Temperatures of 65-75°F (~18-23°C) with 40-60% humidity were applied.
Wild animals	No wild animals were used in the study.
Reporting on sex	Sex as a variable was not tested in these studies design.
Field-collected samples	No field collected samples were used in the study.
Ethics oversight	All procedures were approved by the University of Manchester Animal Welfare Review Body (AWERB) and performed under relevant Home Office licenses according to the UK Animals (Scientific Procedures) Act, 1986.

Note that full information on the approval of the study protocol must also be provided in the manuscript.

Flow Cytometry

Plots

Confirm that:

- [x] The axis labels state the marker and fluorochrome used (e.g. CD4-FITC).
- [x] The axis scales are clearly visible. Include numbers along axes only for bottom left plot of group (a 'group' is an analysis of identical markers).
- [x] All plots are contour plots with outliers or pseudocolor plots.
- [x] A numerical value for number of cells or percentage (with statistics) is provided.

Methodology

Sample preparation	When tumours reached the required endpoint volume, mice were sacrificed by cervical dislocation, and tumours were dissected. Tumours were incubated for 45 minutes with 100 µg/mL of Liberase (Sigma-Aldrich) in serum-free media at 37°C and then pushed through a BD Falcon 100 µM nylon cell strainer using a syringe plunge. The cell suspension was centrifuged at 1300 r.p.m. and 4°C for 7 minutes, and cells were stained for 20 minutes protected from light with LIVE/DEAD™ Fixable Blue Dead Cell Stain Kit (ThermoFisher) diluted at 1:1000 in PBS. Subsequently, Fc receptors were blocked, and cells were stained with surface stain antibody mix for 45 minutes at 4°C in the dark. Cells were fixed and permeabilised using the Foxp3/ Transcription Factor Staining Buffer Set (eBioscience) following the manufacturer's instructions. Intracellular staining was performed for 45 minutes at 4°C in the dark.
Instrument	BD FACSymphony flow cytometer (BD Biosciences)
Software	BD FACSDiva™ software
Cell population abundance	N/A
Gating strategy	Gating strategy are fully elaborated in supplementary figures 4-6.

- [x] Tick this box to confirm that a figure exemplifying the gating strategy is provided in the Supplementary Information.
